# Function of GATA Factors in the Adult Mouse Liver

**DOI:** 10.1371/journal.pone.0083723

**Published:** 2013-12-18

**Authors:** Rena Zheng, Boris Rebolledo-Jaramillo, Yiwei Zong, Liqing Wang, Pierre Russo, Wayne Hancock, Ben Z. Stanger, Ross C. Hardison, Gerd A. Blobel

**Affiliations:** 1 Perelman School of Medicine, University of Pennsylvania, Philadelphia, Pennsylvania, United States of America; 2 Division of Hematology, The Children’s Hospital of Philadelphia, Philadelphia, Pennsylvania, United States of America; 3 Huck Institutes of the Life Sciences, Pennsylvania State University, University Park, Pennsylvania, United States of America; 4 Abramson Family Cancer Research Institute, Perelman School of Medicine, University of Pennsylvania, Philadelphia, Pennsylvania, United States of America; 5 Division of Transplant Immunology, Department of Pathology and Laboratory Medicine and Biesecker Center for Pediatric Liver Disease, Children’s Hospital of Philadelphia, Philadelphia, Pennsylvania, United States of America; 6 Pathology and Laboratory Medicine, The Children’s Hospital of Philadelphia, Philadelphia, Pennsylvania, United States of America; 7 Division of Gastroenterology, Department of Medicine, Department of Cell and Developmental Biology and Abramson Family Cancer Research Institute, Perelman School of Medicine, University of Pennsylvania, Philadelphia, Pennsylvania, United States of America; 8 Department of Biochemistry and Molecular Biology, Pennsylvania State University, University Park, Pennsylvania, United States of America; Università degli Studi di Milano, Italy

## Abstract

GATA transcription factors and their Friend of Gata (FOG) cofactors control the development of diverse tissues. GATA4 and GATA6 are essential for the expansion of the embryonic liver bud, but their expression patterns and functions in the adult liver are unclear. We characterized the expression of GATA and FOG factors in whole mouse liver and purified hepatocytes. GATA4, GATA6, and FOG1 are the most prominently expressed family members in whole liver and hepatocytes. GATA4 chromatin immunoprecipitation followed by high throughput sequencing (ChIP-seq) identified 4409 occupied sites, associated with genes enriched in ontologies related to liver function, including lipid and glucose metabolism. However, hepatocyte-specific excision of *Gata4* had little impact on gross liver architecture and function, even under conditions of regenerative stress, and, despite the large number of GATA4 occupied genes, resulted in relatively few changes in gene expression. To address possible redundancy between GATA4 and GATA6, both factors were conditionally excised. Surprisingly, combined *Gata4,6* loss did not exacerbate the phenotype resulting from *Gata4* loss alone. This points to the presence of an unusually robust transcriptional network in adult hepatocytes that ensures the maintenance of liver function.

## Introduction

GATA transcription factors typically function in concert with Friend of GATA (FOG) cofactors to regulate the formation of numerous cell types [1,2] . The GATA family is comprised of six members that all possess a highly conserved double zinc finger domain that mediates binding to DNA and to co-factors such as FOG proteins [3]. GATA factors are expressed in distinct patterns: GATA1, GATA2, and GATA3 mainly in the hematopoietic system and GATA4, GATA5, and GATA6 in endoderm and mesoderm derived tissues [4,5]. FOG1 and FOG2 are multi-type zinc finger proteins that like GATA factors are expressed in a highly tissue-restricted fashion [1]. FOG proteins are unable to bind DNA directly and function entirely via association with the N-terminal zinc fingers of GATA factors [6,7]. Both GATA1 and FOG1 are essential for normal erythropoiesis [6,8-10] and megakaryopoiesis [11] in a manner dependent upon their direct physical interaction [12-14]. Similarly, direct contacts between GATA4 and FOG factors are required for the development of the heart and small intestine [15,16]. 

GATA factors play a key role in the development of the liver. The liver develops from the ventral foregut endoderm in close proximity to the cardiogenic mesoderm [17]. Inductive mesoderm signals, including signaling from fibroblast growth factor (FGF) family members, are crucial for specification of hepatic fate [17,18]. GATA4 and GATA6 are each important for the expansion of the liver bud during embryogenesis, but serve redundant functions in hepatic specification [19,20]. GATA4 expression is first detected in the ventral foregut endoderm and cardiac mesoderm at the 4 somite stage (embryonic day E8.0) [19]. At the 16 somite stage, GATA4 expression is still detected in the foregut endoderm, which includes the liver bud and surrounding septum transversum mesenchyme, but is extinguished selectively from hepatoblasts at the 25 somite stage (E10.0) [19-21], suggesting a highly developmentally restricted expression of GATA4 only during the earliest stages of hepatocyte specification. During this window, GATA4 is required for the full expression of select hepatic genes, including albumin and hepatocyte nuclear factor 4 (HNF4) [19]. GATA6 expression is detected in the ventral foregut endoderm at the 6-8 somite stage [20]. By the 12 somite stage, GATA6 is detected in the liver bud and septum transversum and in contrast to GATA4, persists to the 25 somite stage in hepatocytes [20]. While GATA4 and GATA6 appear to each be required for liver bud expansion, whether this function is hepatocyte cell-autonomous has not been fully explored [19,20]. Loss of function studies in developing zebrafish [22] and Xenopus [23] have confirmed that the role of GATA4 and GATA6 in liver development is conserved across vertebrates. However, a clear picture of GATA factor function and their transcriptional programs in mature adult hepatocytes is lacking. 

GATA6 expression has been reported in fetal and adult hepatocytes as well as adult cholangiocytes [20,21,24]. Reports on the predominant sites of GATA4 expression in the adult liver vary significantly and include hepatocytes [25], endothelial cells [20], and cholangiocytes [26]. The reasons for these disparities are unclear but might be rooted in the sensitivity and specificity of antibodies used for immunohistochemistry. This leaves open the question as to what role, if any, GATA4 plays in adult hepatocytes *in vivo*. 

Studying liver-related functions of GATA4 and/or GATA6 in knockout mice has been hampered by early embryonic lethality unrelated to adult liver function [27-29]. In transformed hepatoma cell lines, GATA4 activates liver-specific genes encoding coagulation factor X [30], homeobox gene *Hex* [31], a cytochrome p450 [32], and hepcidin [33]. However, upon culture, primary hepatocytes as well as hepatoma cells undergo substantial changes in gene expression patterns, including diminished production of key liver transcription factors such as C/EBPα and FOXA1 (also known as HNF3α) [34]. GATA4 has also been implicated in the expression of the hepatocyte expressed albumin gene based on footprinting experiments in the ventral foregut endoderm from which the embryonic liver forms [35]. Moreover, GATA4 together with HNF3/FOXA binds in vitro to the albumin gene embedded in nucleosomal arrays to form a nucleosome free region [36]. Together these findings implicate GATA4 in control of gene expression in hepatocytes, but whether GATA4 functions in primary adult hepatocytes to control liver-specific gene expression remains an open question. 

Additional circumstantial evidence for GATA factor function in the adult liver derives from our studies of mice homozygous for FOG1 point mutations that disrupt the interaction between FOG1 and the chromatin remodeling complex NuRD. In addition to hematopoietic defects [37-39] emerging preliminary data suggest that mutant mice aged beyond one year have an increased propensity for hepatocellular carcinoma (HCC) (Figure S1). This raises the possibility that GATA, FOG1, and NuRD proteins function together as tumor suppressors in hepatocytes, providing an additional incentive to study the normal functions of GATA and FOG proteins in hepatic gene expression.

We find that GATA4, GATA6, and FOG1 are expressed in whole mouse liver and purified primary hepatocytes. Using chromatin immunoprecipitation followed by massive parallel sequencing (ChIP-seq), and conditional excision we characterize in-depth the GATA4-controlled transcriptional program in the adult liver. We found that GATA4 occupies over 4000 sites in the genome. The ontologies of sites associated with these genes relate to liver specific functions. Conditional excision of *Gata4* or both *Gata4* and *Gata6* from hepatocytes yielded a surprisingly mild hepatic phenotype. This suggests the presence of an unusually robust transcriptional network that stably maintains hepatocyte-specific gene expression patterns even in the absence of two GATA factors.

## Materials and Methods

### Mice

*Fog1 ki/ki* mice were previously described [38]. Wildtype and *Fog1 ki/ki* mice were maintained on a mixed C57BL/6xSv129 background. *Gata4 fl/fl* (stock# 008194) mice were purchased from Jackson Laboratories. *Gata4,6 fl/fl* mice were a generous gift from Stephen Duncan (Medical College of Wisconsin). Genotyping primers are listed in table S1. This study was carried out in strict accordance with federal guidelines and institutional policies. The protocol was approved by the Institutional Animal Care and Use Committee of the Children’s Hospital of Philadelphia (Protocol# 2012-7-660).

### Hepatocyte isolation

Mice were anesthetized with inhaled isoflurane (Baxter). The abdominal cavity was opened and inferior vena cava (IVC) was exposed. An angiocath was inserted into the IVC and HBSS with 1 mM EGTA, followed by HBSS with 5 mM CaCl_2_ and 40 µg/ml Liberase (Roche), were pumped into the IVC using a variable flow mini pump (Fisher Scientific). Liver tissue was dissociated in hepatocyte wash medium (GIBCO) and filtered through a 100 µm membrane. To isolate the hepatocyte fraction, the single cell suspensions were mixed 1:1 with Percoll Plus solution (GE Healthcare) and spun at 120 x g. To isolate “all cells” (all cells that have undergone collagenase digestion but not gradient centrifugation) the single cell suspension was spun in hepatocyte wash medium alone at 120 x g. ACK lysing buffer (Lonza) was used to lyse potentially contaminating erythrocytes. Cells were either directly placed in Trizol to proceed with RT-qPCR or fixed in formaldehyde to proceed with ChIP. 

### RT-qPCR

RNA was isolated using TRIzol reagent (Invitrogen) from 0.1 mg whole liver or 0.5x10^5^-1x10^6^ cells from all liver cells or hepatocytes. Reverse transcription reactions were performed using Superscript II (Invitrogen). cDNA was quantified by Power Sybr Green qPCR. Data were normalized to beta actin and GAPDH, with no major differences. Data in the figures show expression relative to actin. Relative expression was calculated as 2^CT actin – CT primer^. RT-PCR primers are listed in table S2.

### ChIP

ChIP on whole liver, all cells and hepatocytes was performed as described previously [40]. “Whole liver” describes intact livers processed directly. “All cells” refers to single cell suspensions encompassing all liver cell types that have undergone collagenase digestion, but not Percoll gradient centrifugation. “Hepatocytes” are cells that have been collagenase digested and undergone Percoll gradient centrifugation. Anti-GATA4 C-20, goat polyclonal IgG (Santa Cruz), was used for all GATA4 ChIP experiments. An additional anti-GATA4 H-112, rabbit polyclonal IgG (Santa Cruz), was used for one GATA4 ChIP experiment as listed on figure. ChIP-qPCR primers are listed in table S3.

### Western analysis

Nuclear extracts were made from 5-10x10^6^ hepatocytes, all liver cells, 293T cells and derivatives, or JC4 erythroid cells and from 40-100 mg of whole liver or heart. 100 μg of protein for whole liver, hepatocytes or all liver cells and 1-25 μg of protein for controls were run on a 10% SDS-PAGE gel followed by transfer to a nitrocellulose membrane. Antibodies used for western blotting are anti-GATA4 C-20 (Santa Cruz), anti-FOG1 M-20 (Santa Cruz) and anti-beta actin-peroxidase (Sigma). 

### AAV-Cre and AAV-GFP injection in GATA4 fl/fl mice

*Gata4 fl/fl* mice were intravenously injected with 1.5x10^11^ virus particles of either AAV8-Tbg-Cre (AV-8-PV1091) or AAV8-Tbg-GFP (AV-8-PV0146), purchased from Penn Vector Core. Two weeks post injection, hepatocytes were isolated and RT-qPCR, ChIP or western was performed.

### Microarray

RNA purification was carried out using Trizol reagent (Invitrogen) according to manufacturer’s recommendations. RNA quality was determined using the Bioanalyzer (Agilent). Only samples with RNA integrity number (RIN) >7.5 were used for further studies. Equal amounts (100ng) of total RNA were amplified with Epicentre TargetAmp™- Nano Labeling Kit for Illumina® Expression BeadChip and hybridized to the MouseWG6v2 mouse whole genome bead arrays. Illumina GenomeStudio software was used to export expression levels and detect p-values for each probe of each sample. Arrays were quantile-normalized and filtered to remove non-informative probes (probes with a detection p-value>0.05 in all samples). A list of significant genes differentially expressed between two classes of samples was determined by using SAM method [41] with false discovery rate (FDR) threshold set at FDR<20%, <25%, <26%, or <30% (as indicated on figure). Identification of biological functions and pathways overrepresented in any gene list was done using Ingenuity Pathway Analysis (IPA) software (Ingenuity Systems, Redwood City, CA). IPA results were filtered to satisfy FDR <25% criteria unless stated otherwise. 

### Library preparation and high-throughput sequencing

ChIP was performed using anti-GATA4 C-20 (Santa Cruz) on two biological replicates, each consisting of whole liver from two mice (age 2.5 to 3.5 months). For sequencing libraries, the input (liver) and ChIP DNA fragments were repaired to generate blunt ends, with a single adenine added to each end. Illumina genomic adaptors were ligated to both ends of the fragments, ligation products were amplified by 18 cycles of PCR, and the PCR products between 250 and 450 bp were gel purified according to standard Illumina protocols. The quantity and quality of each library was evaluated by qPCR and Bioanalyzer (Agilent Technologies, Santa Clara, CA). Cluster generation and sequencing chemistry were performed using Illumina-supplied kits as appropriate. The ChIP DNA library was sequenced in single-read mode on the Illumina HiSeq 2000 platform.

### Reads mapping

47.9 million (M) (replicate 1) and 47.6M (replicate 2) GATA4 ChIP-seq, and 60.5M (replicate 1) and 53.1M (replicate 2) INPUT 48-bp sequencing reads were aligned to the canonical reference mouse genome (Build 37, mm9) using bowtie v.0.12.7 [42] on Galaxy (www.usegalaxy.org). Only uniquely mapped reads, representing roughly 65% of the total number of reads per sample, were kept for further analyses.

MACS v1.3 [43] implementation on Galaxy (www.usegalaxy.org) was used to generate BigWig (http://genome.ucsc.edu/goldenPath/help/bigWig.html) signal tracks.

### Peak calling

Peaks (“occupied sites”) were called using a modified version of the SPP peak caller [44] and MACS v2.0.10 [43] following the Irreproducibility Discovery Rate (IDR) protocol described by Anshul Kundaje (9/28/2012, IDR: Reproducibility and automatic thresholding of ChIP-seq data. In IDR: Reproducibility and automatic thresholding of ChIP-seq data. Retrieved 11/13/2012, from https://sites.google.com/site/anshulkundaje/projects/idr), which is based on the reproducibility method developed by Qunhua Li and Peter Bickel [45]. Briefly, low stringency peaks were called with both SPP and MACS v2.0.10 on the two GATA4 ChIP-seq replicates and a pooled sample generated by merging both ChIP-seq replicates mapped reads. The peaks were called against the pooled input mapped reads as background. The IDR method requires low stringency peaks to test reproducibility among the replicates. It compares the rank of a particular peak region in a sorted list (SPP peaks were sorted by signal value, while MACS v2.0.10 peaks were sorted by p-value), splits true peaks from noise, and defines a set of peak regions whose rank is concordant in both replicates at a given threshold. Details of the protocol and thresholds used can be found at https://sites.google.com/site/anshulkundaje/projects/idr.

SPP peaks (4811 regions) were intersected with the MACS 2.0.10 peaks (5867 regions) using BedTools v2.16.1 [46], and defined a “common” set of peaks (4409 regions, retaining SPP’s coordinates) for further analyses.

### Genomic distribution

Build 37 (mm9) mouse reference gene coordinates were obtained from the table "RefGene" on the UCSC table browser (http://genome.ucsc.edu). The transcription start site (TSS), transcription termination site (TTS) and exons coordinates were extracted from the table to define the following custom intervals in BED format: (1) 5 kb upstream of TSS to the end of first exon, (2) first intron, (3) rest of gene, and (4) last exon to 5 kb downstream of TTS. Any peak outside the range TSS-5kb, TTS+5kb was deemed intergenic. An additional set of custom intervals substituting 10 kb in the above definitions was also used, as indicated in figure and text. The 4409 “common” peaks were intersected with the custom intervals using BedTools v2.16.1 [46] to generate pie charts. Peaks were allowed to intersect multiple custom intervals because we observed that in some cases the proximity between “gene A” and “gene B” would generate overlapping categories, i.e. downstream region of “gene A” overlapped the upstream region of “gene B”. Consequently, the percentages shown in the pie charts were calculated by dividing the number of peaks falling in a particular category by the total number of intersections, rather than the total number of peaks.

### Motif discovery

Motif discovery and enrichment was conducted with the motif discovery tool suit MEME ChIP v.4.9.0 [47], available at http://meme.nbcr.net/. Default parameters were used, except for the following: database = Jolma2013, count of motifs = 10, min. width = 6 and max, width = 6. FIMO (Find Individual Motif Occurrences, a part of the MEME suit of tools) was used to scan the peak sequences for occurrences of the WGATAR motif. The distribution of the strength of the underlying signal (SPP's tag enrichment score) of peaks with and without the WGATAR motif was used to generate boxplots.

### Heart and liver GATA4 occupied segments intersection

Heart GATA4 occupied segments genomic coordinates were obtained from He et al, 2011[48]. The intersection between the heart set and our set of 4409 liver GATA4 occupied segments was calculated with BedTools v2.16.1 [46].

### Genome Regions Enrichment of Annotations Tool (GREAT) analysis

GREAT analysis [49] was performed on the 4409 liver GATA4 ChIP-seq binding sites, the 956 binding sites common to liver and heart GATA4 ChIP-seq binding sites, and the 3453 unique liver GATA4 binding sites using the website http://bejerano.stanford.edu/great/public/html/. Association rule settings were to single nearest gene with an extension of no greater than 50 kb from transcriptional start site, including curated regulatory domains.

### Intersection of intergenic peaks with mouse ENCODE enhancer histone marks

Data were analyzed and peaks assigned using two windows spanning either 5 kb or 10 kb upstream of the transcription start site (TSS) and downstream of the transcription termination site (TTS). Peaks outside these ranges were defined as intergenic. Mouse ENCODE’s eight week old mouse liver H3K4me1 and H3K27ac histone marks peaks were obtained from the UCSC genome browser (http://genome.ucsc.edu), and intersected with the set of intergenic peaks with BedTools v2.16.1 [46]. 

### Accession Number

The ChIP-seq and microarray datasets have been deposited in a superseries in GEO Database under the accession number GSE49132. 

### Liver Histology

The hematoxylin-eosin (HE) stains were performed using 5 µm thick sections from formalin-fixed, paraffin-embedded tissue blocks. Specimens were fixed in formalin (Fisher) immediately after harvesting and followed by gradient dehydration with 50%, 70%, 95%, and 100% ethanol. Tissue were then processed in xylene (Fisher) and embedded in paraplast tissue embedding medium (Fisher). Slides were prepared using Microm HM550. Paraffin sections were deparaffinized in xylene followed by rehydration with 100%, 95%, 70% ethanol and then Millipore water before the staining. Harris hematoxylin (Fisher) was used for nuclei staining. Excess hematoxylin was removed by dipping slides in acid alcohol (Leica Biosystems Richmond, 3560). Slides were then placed in running warm water until the nuclei turned blue. Eosin (Leica Biosystems Richmond, 1600) was used to stain for cytoplasm. Slides were later mounted using permount (Fisher, SP15-500) after clearing with xylene. Digital images of slides were taken on Axioskop 2 microscope (Zeiss).

### Serum studies

Blood was obtained from the heart and allowed to coagulate at room temperature for one hour, then spun at 10,000 rpm for 10 min. Serum was removed to a fresh tube and analyzed with a Vitros 350 Chemistry Analyzer (Johnson & Johnson). 

### Partial hepatectomy

A mouse was anesthetized by inhaled isofluorane. An incision was made down the midline of the abdomen and the left and middle lobes of mouse liver were exposed. The falciform ligament was cut down to the superior vena cava and the membrane linking the caudate and left lobes was also cut. A 1-0 suture was placed at the bottom of the left and middle lobes. The ends of the suture were tied tightly and the lobes were cut. After determining that no bleeding occurred, the abdominal incision was closed with a 4-0 suture in one layer.

## Results

### GATA and FOG expression in whole liver and isolated hepatocytes

**Figure 1:**
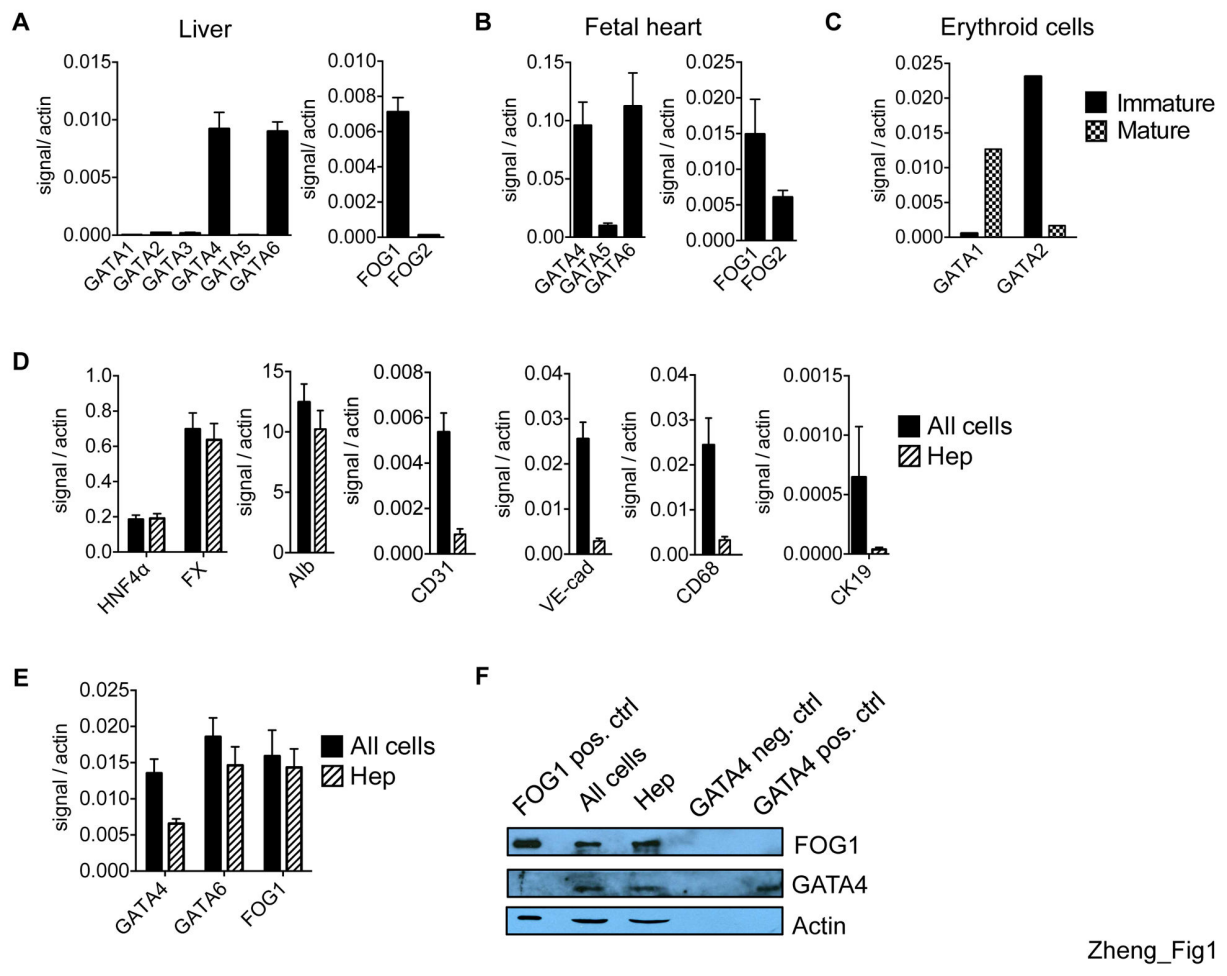
GATA and FOG expression in whole liver and isolated hepatocytes. Relative mRNA levels of GATA and FOG family members as measured by RT-qPCR and normalized to β actin in (A) liver (n=5), (B) fetal heart (n=4), and (C) immature and mature erythroid cells. (D) Relative mRNA levels in all liver cells (all cells, black bars) and hepatocytes (hep, striped bars) of hepatocyte expressed genes (HNF4α, factor X (FX), albumin (alb)), endothelial specific genes (CD31,VE-cadherin (VE-cad)), Kupffer cell specific gene CD68 and cholangiocyte specific gene CK19. (E) GATA4, GATA6, and FOG1. (n=15 in all cells and hep for all primers except n=3 for CK19) (F) Western blot of GATA4 and FOG1 proteins with actin loading control. Erythroid cells served as positive control for FOG1. 293T cells and derivatives expressing GATA4 served as positive (pos. ctrl) and negative controls (neg. ctrl), respectively.

We examined the expression of GATA and FOG mRNAs in whole adult mouse livers by quantitative RT-PCR. GATA4 and GATA6 are the only significantly expressed members of their family (Figure 1A) with levels approximating one-tenth of those observed in the fetal heart, which served as positive control (Figure 1B). FOG1 but not FOG2 is expressed at significant levels reaching up to one half those found in the fetal heart (Figure 1B). Immature and mature erythroid cells [50] served as positive controls for GATA2 and GATA1, respectively (Figure 1C). Although the liver is composed of ~70% hepatocytes [18], previous studies have also implicated endothelial cells [20] and cholangiocytes [26] as the source of GATA4 in adult liver. Inconsistencies among published immunohistochemical (IHC) analyses are likely rooted in variations of sensitivities and specificities of antibodies as well as tissue preparations. Moreover, IHC is usually not very quantitative. In our hands, IHC experiments provided ambiguous results as to the expression of GATA4 in hepatocytes (data not shown), perhaps in part due to the relatively low levels of expression when compared to the fetal heart. To determine GATA and FOG expression levels in primary hepatocytes versus non-hepatocyte lineages by an independent means, we purified hepatocytes from whole livers by collagenase digestion and density gradient centrifugation, a well established procedure for isolating hepatocytes [51,52]. As a control we also collected an “all cells” fraction containing all liver cell types that had gone through the collagenase digestion protocol but were not subjected to gradient centrifugation. Purity of the hepatocyte population was reflected in the presence of hepatocyte-specific transcripts HNF4α (hepatocyte nuclear factor 4α), coagulation factor X (FX), and albumin at levels comparable to all cells, and the depletion of endothelial-cell specific transcripts CD31 and VE-cadherin, and the Kupffer cell-specific mRNA CD68 (Figure 1D). To assess the possible contamination by cholangiocytes of the hepatocyte fractions, we compared the expression of the cholangiocyte-specific genes CK19 [53] by qRT-PCR and observed a significant depletion of these transcripts in hepatocytes (Figure 1D). Thus, our preparations were highly enriched for hepatocytes. We next examined GATA and FOG factor expression in purified hepatocytes. Notably, GATA4 was expressed at significant levels amounting to approximately half of those observed in all cells (Figure 1E). However, since hepatocytes represent the predominant proportion of cells in the liver, this implies that non-hepatocytes, although underrepresented, contribute a substantial proportion of GATA4 expression in the liver. Despite the low level of GATA4 expression, GATA4 has high chromatin occupancy at target genes (see below and Figure S2A). The expression of GATA6 and FOG1 in hepatocytes is comparable to that of all cells, suggesting that hepatocytes are a main source of these factors (Figure 1E). We also detected GATA4 and FOG1 proteins by Western blotting in nuclear extracts from both all cells and purified hepatocytes (Figure 1F). GATA6 protein could not be detected by Western blotting due to lack of a sensitive GATA6 antibody. In conclusion, our results indicate that GATA4, GATA6, and FOG1 are the predominantly expressed members of the GATA and FOG families in adult hepatocytes.

### GATA4 occupies hepatocyte-specific target genes

**Figure 2:**
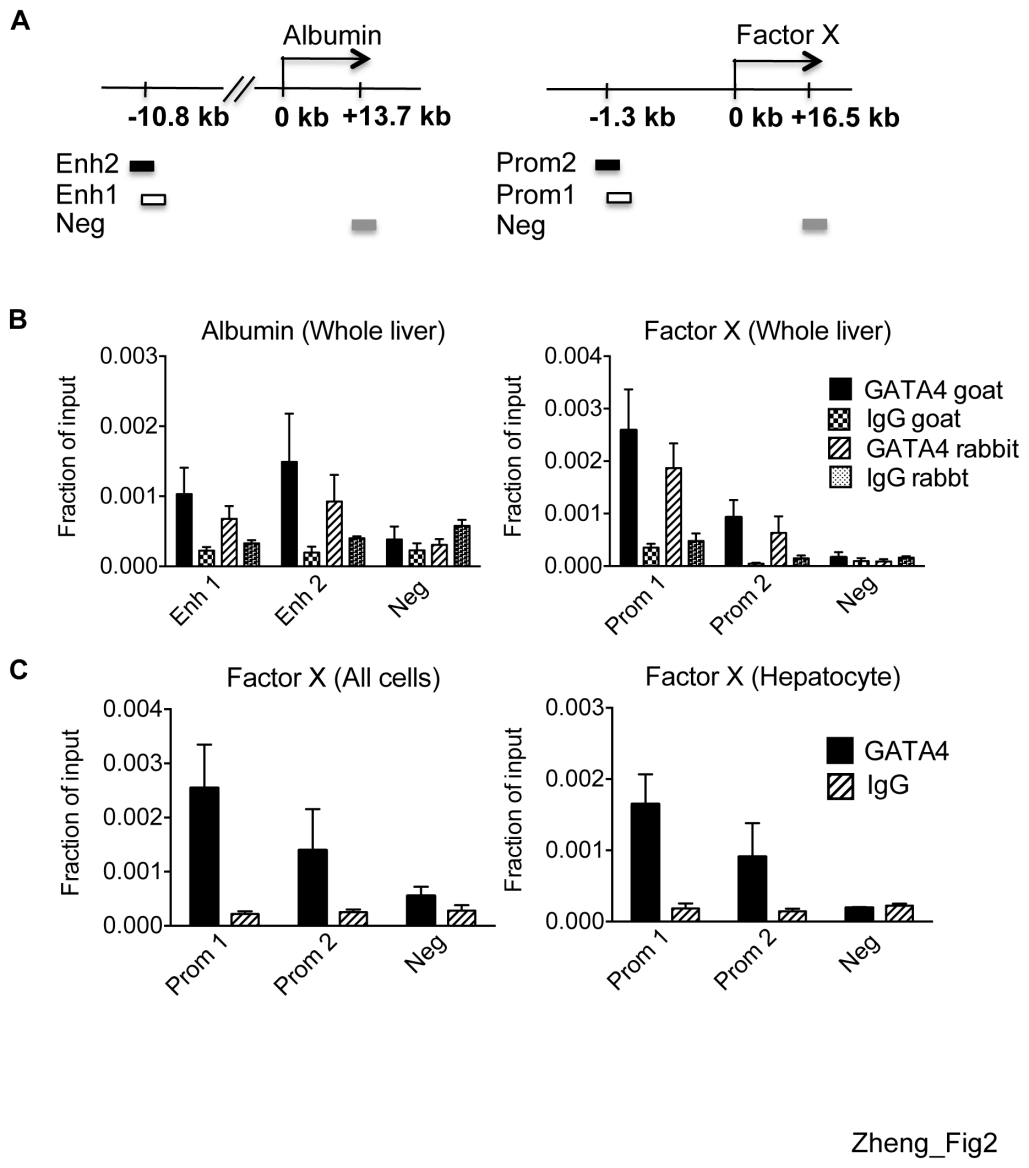
GATA4 occupies targets in whole liver and isolated hepatocytes. (A) Schematic of the albumin (left) and factor gene (right). “0” denotes the TSS. Boxes represent ChIP-qPCR primer amplicons. Enh, enhancer; prom, promoter, and neg, negative control. (B) GATA4 ChIP-qPCR in whole livers at albumin (left) and factor X (right) genes (n=3). (C) GATA4 ChIP-qPCR at factor X in all cells that have undergone collagenase digestion but not gradient centrifugation (left) and hepatocytes (right) (n=3-5).

GATA4 occupancy at select hepatocyte-specific genes has been described in cell lines [20,21,24,30,31,33] and in vitro on nucleosomal arrays [36] but whether GATA4 occupies hepatocyte-specific target genes in primary cells *in vivo* is unknown. We performed chromatin immunoprecipitation (ChIP) with two independent anti-GATA4 antibodies followed by qPCR to assess GATA4 binding to presumed GATA4 target genes in whole liver preparations. Primer pairs were designed to span two sequences each at the albumin enhancer and factor X promoter upstream region, according to previously described GATA4 target sites [30,36]. In addition, a negative control site lacking the WGATAR sequence was included at each of these genes (Figure 2A). GATA4 occupancy was detected at the albumin enhancer and factor X promoter (Figure 2B). We next performed anti-GATA4 ChIP on purified hepatocyte populations and, as a control, the all cells population. GATA4 occupancy was confirmed at the factor X promoter (Figure 2C) and additional target genes (see below) at comparable levels in hepatocytes and all cells. Together, these results demonstrate that GATA4 occupies hepatocyte-specific genes *in vivo*, suggesting a role for GATA4 in regulating hepatic functions in the adult liver.

### Genome wide GATA4 occupancy analysis reveals liver specific targets

**Figure 3:**
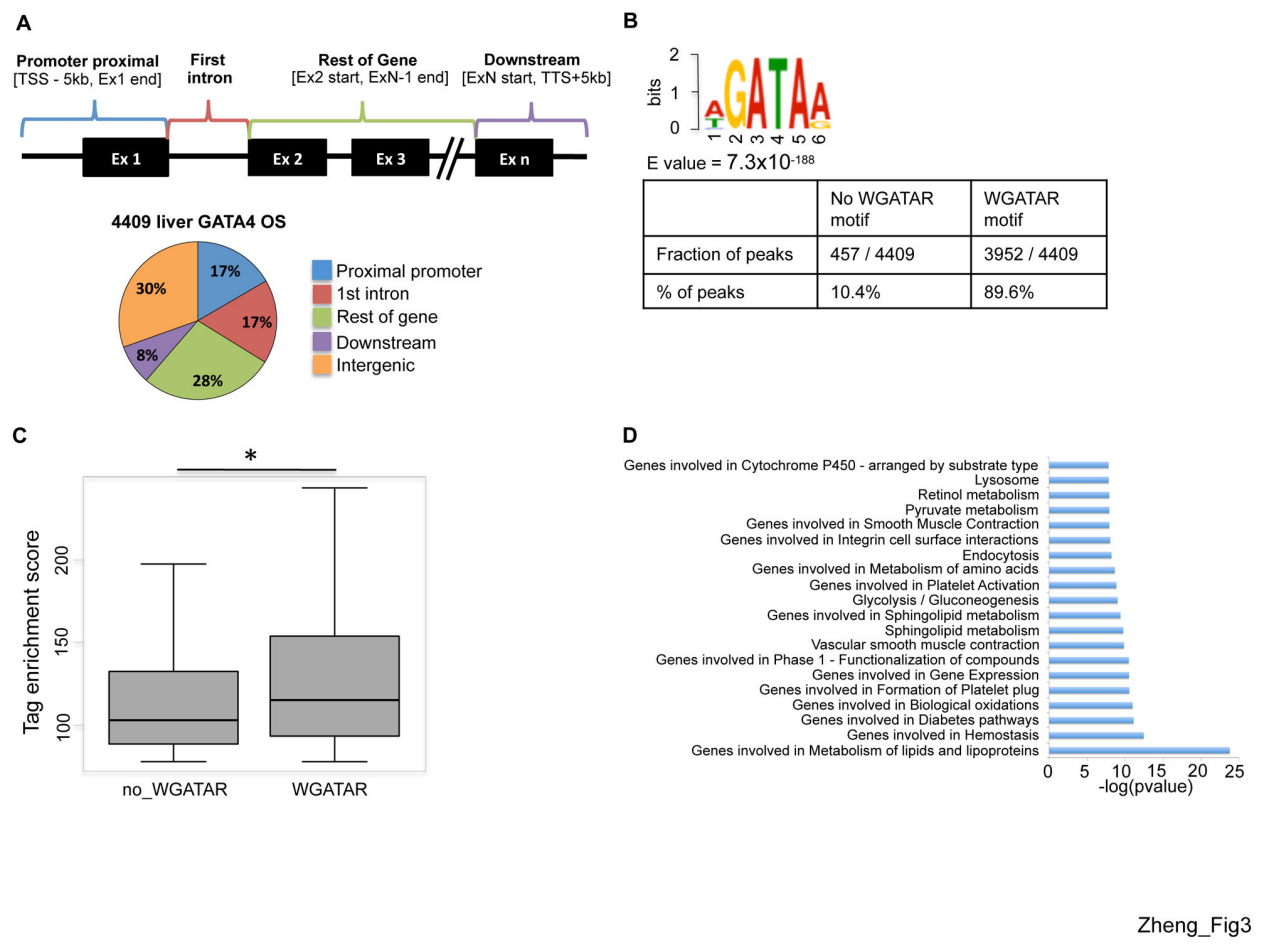
GATA4 ChIP-seq in the adult liver. (A) Distribution of GATA4 occupied sites: promoter proximal: 5 kb upstream of the TSS to the end of first exon (blue); first intron (red); rest of gene (second exon until penultimate exon, green); downstream (last exon to 5 kb downstream of TTS, purple), and intergenic (any region outside of all of the above, orange). (B) MEME analysis identified WGATAR motifs as most enriched under GATA4 OS. Table summarizing fractions and percentages of GATA4 OS with and without WGATAR motifs. (C) Box and whiskers plot of ChIP-seq tag enrichment scores (y axis) between peaks with and without WGATAR motif, * p = 1.258x10^-5^, Student’s T test. (D) Functional ontologies of GATA4 occupied genes as assigned by GREAT with Molecular Signatures Database (MSigDB).

In order to reveal the transcriptional program under the control of GATA4 in the adult liver, we performed GATA4 ChIP with anti-GATA4 C-20 antibody (Santa Cruz) followed by high throughput sequencing (ChIP-seq) in whole adult mouse liver. Genomic DNA not subjected to immunoprecipitation was sequenced as input control. Using two independent peak calling methods and irreproducible discovery rate (IDR) analysis [45], we identified 4409 GATA4 occupied sites (OS), of which 2918 OS bind 3075 unique genes when using a window of 5kb upstream of the transcription start site (TSS) of genes to 5kb downstream of the transcription termination site (TTS). If this window is extended to 10 kb upstream of the TSS and downstream of the TTS, 3168 GATA4 OS fall within 3694 genes. We intersected 1491 intergenic GATA4 occupied sites outside the 5kb (Figure S3C) window with publically available datasets of H3K4me1 and H3K27ac ChIP-seq, which are markers of enhancers [54]. Approximately 40% of GATA4 intergenic sites outside of 5kb from genes are associated with these enhancer marks. Using the 10kb window leaves 1241 intergenic sites of which 40% also have these enhancer marks (Figure S3D). 19 out of 19 GATA4 OS from a spectrum of IDR significance levels were detected by ChIP-qPCR, validating the ChIP-seq results and peak calling methods (Figure S2A). In addition, a strong GATA4 OS signal was observed at the factor X promoter confirming ChIP-qPCR results from Figure 2 (Figure S2B). When examined at the 5 kb window described above, 17% of GATA4 OS are at the promoter proximal regions, 17% fall within the first intron while 28% are located within the coding regions of target genes and 8% are within the last exon and regions proximal to the TTS (Figure 3A). The remaining 30% of GATA4 OS are intergenic. This occupancy profile is very similar to that obtained when the window is extended to 10 kb upstream and downstream, respectively of TSSs and TTSs (Figure S3A). To determine sequence motifs enriched at the GATA4 OS, we performed MEME motif analysis [47]. The canonical GATA motif, (A/T)GATA(A/G) or WGATAR, was the most highly enriched sequence and found in almost 90% of the occupied sites (Figure 3B). This percentage rises to 95% if the non-canonical GATA motifs WGATA or GATAR are included (Figure S4). In addition, ChIP-seq tag enrichments are higher at GATA4 OS containing the WGATAR motif compared to GATA4 OS lacking the WGATAR motif (Figure 3C). Together, these results indicate that GATA4 occupies a substantial number of sites via direct DNA binding in the adult liver.

**Figure 4:**
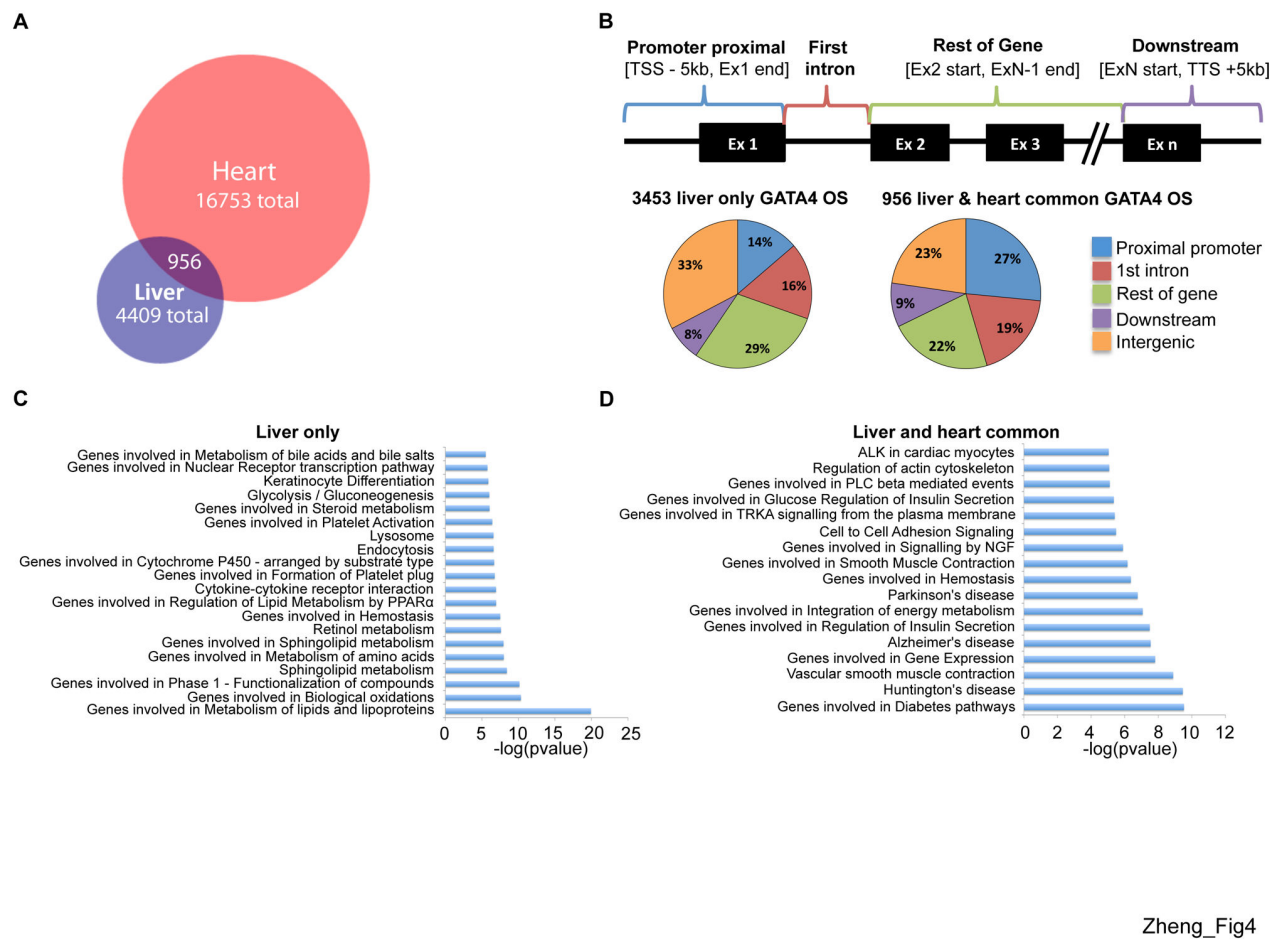
The great majority of GATA4 occupied sites in the liver are liver specific. (A) Intersection of liver and heart GATA4 ChIP-seq data sets. (B) Distribution of GATA4 occupied sites (see Fig. 3 for explanation) in liver-only (left) and liver and heart common occupied sites (right). GREAT analysis assigning functional ontology terms from the Molecular Signatures Database (MSigDB) to GATA4 occupied genes in (C) liver-only and (D) liver and heart common datasets.

To further explore the functional significance of liver GATA4 OS, we used the Genome Regions Enrichment of Annotations Tool (GREAT) [49] to assign GATA4 ChIP-seq peaks to single genes within a gene regulatory region between midpoints of consecutive TSS, with maximum extension of no more than 50 kb in one direction from the TSS. Two thirds of the peaks were assigned to single genes according to these criteria. Notably, these genes belonged to ontological groups related to liver function, including lipid metabolism, glycolysis/gluconeogenesis and phase 1 functionalization of compounds (cytochrome p450 mediated metabolism) (Figure 3D). To delineate gene expression programs under the control of GATA4 that are specific to the liver we compared the liver ChIP-seq data set with a previously reported dataset of genome-wide GATA4 occupancy sites in cardiomyocyte cell lines [48]. Intersection of these datasets revealed that 20% of the liver GATA4 OS overlap with those from heart whereas 80% of liver GATA4 OS are liver specific (Figure 4A). The distribution pattern of liver-only sites (Figure 4B left) closely matched that of the entire liver (Figure 3A) and heart-only sites (Figure S3B), but the distribution of the common binding sites showed greater enrichment in promoter proximal regions (Figure 4B right). GREAT analysis of the liver-only GATA4 OS revealed ontologies related to metabolism of lipids, bile acid, and amino acids, glycolysis/gluconeogenesis, and phase 1 functionalization of compounds, similar to the ontologies from the entire liver GATA4 ChIP-seq dataset (Figure 4C). However, the ontologies of genes associated with GATA4 OS that are shared between liver and heart were more diverse, with the majority not being liver related (Figure 4D). In concert, these analyses suggest a role for GATA4 in adult liver gene expression. 

### Conditional Gata4 deletion from hepatocytes of adult mouse liver

**Figure 5:**
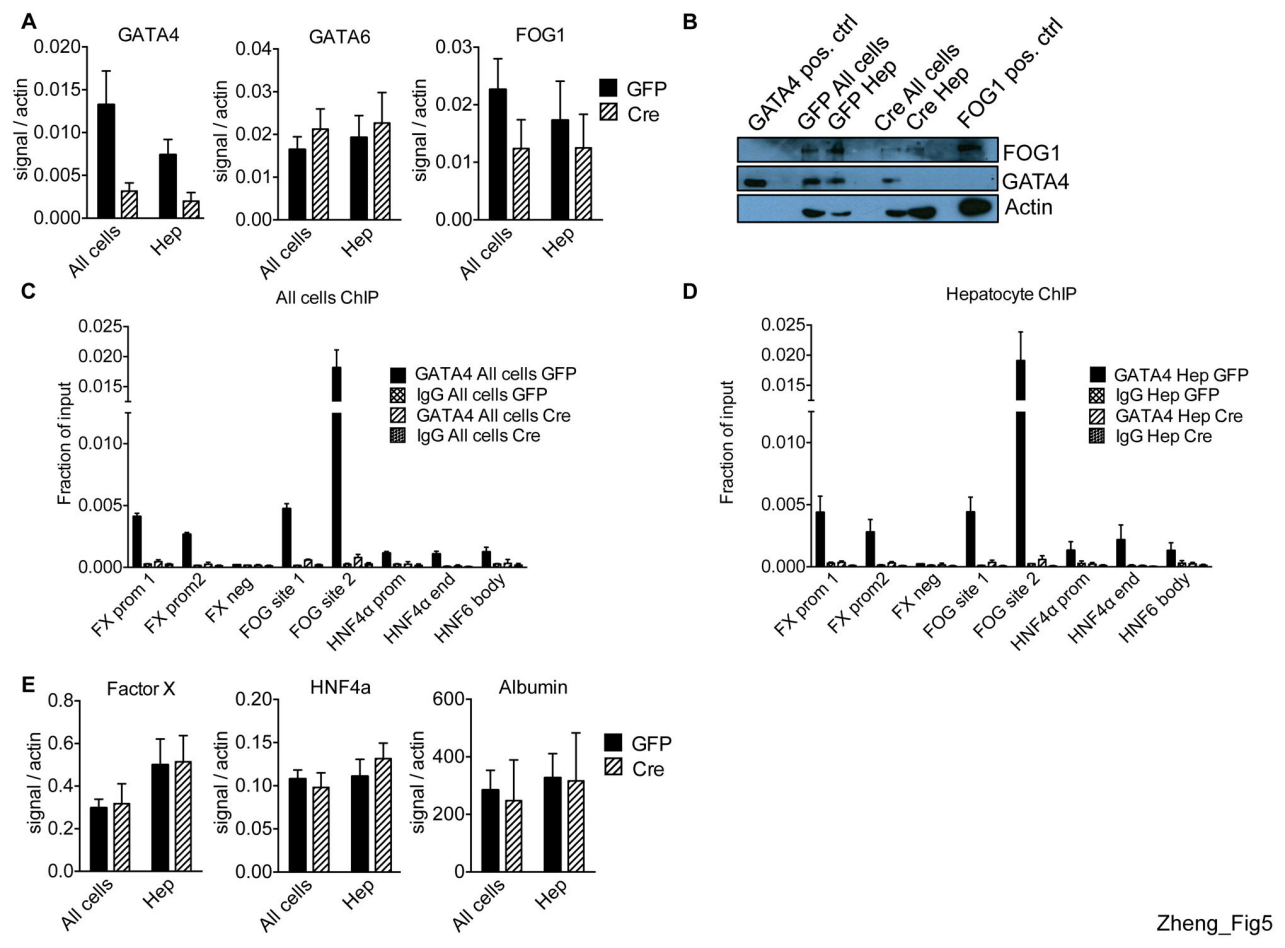
GATA4 excision in hepatocytes. (A) mRNA levels of GATA4, GATA6 and FOG1 in all liver cells (all cells) and hepatocytes (hep) of AAV-GFP (black bars) and AAV-Cre (striped bars) injected mice (n=5-7). (B) Western blot of GATA4 and FOG proteins. Heart and erythroid cells served as positive controls (pos. ctrl) for GATA4 and FOG1, respectively. GATA4 ChIP-qPCR in all cells (C) and hep (D) (n=2-3) at factor X (FX) promoter (prom) and negative control region (neg), FOG1, HNF4α promoter and 3’ end of gene (end) and HNF6 intragenic (body) sites (n=2-3). (E) mRNA levels of factor X, HNF4α, and albumin in All cells, Hep of AAV-GFP (black bars) or AAV-Cre (striped bars) injected mice (n=4).

To examine the function of GATA4 in the mature liver we deleted it selectively in hepatocytes of adult mice. Animals homozygous for floxed *Gata4* alleles (*fl/fl*) were injected via tail veins with adeno-associated virus expressing Cre recombinase (AAV-Cre) or, as control, GFP (AAV-GFP) from the hepatocyte-specific thyroxine-binding globulin (Tbg) promoter [55]. Two weeks after AAV-Cre injection, GATA4 mRNA and protein levels were markedly reduced from hepatocytes and all cells, indicating successful excision of *Gata4* (Figure 5A,B). The residual GATA4 mRNA expression in hepatocytes is most likely due to background signal, as GATA4 protein level (Figure 5B) and occupancy (Figure 5C and 5D, discussed below) are completely depleted. GATA6 levels remained steady in all cells and hepatocytes of AAV-Cre and AAV-GFP injected *Gata4 fl/fl* mice suggesting a lack of cross-regulation among these factors and contrasting with the cross-regulation of GATA factors in hematopoietic cells [56]. FOG1 levels were slightly reduced in hepatocytes and all cells of AAV-Cre injected mice, suggesting that FOG1 is a GATA4 regulated gene (Figure 5A and 5B). Indeed, GATA4 strongly occupies the FOG1 locus, indicating that FOG1 is a direct GATA4 target (peak 19 and peak 20, Figure S2A), reminiscent of FOG1 regulation in erythroid cells by GATA1 [13]. Importantly, when examined by ChIP, deletion of *Gata4* resulted in a virtually complete loss of GATA4 from all examined sites, factor X, FOG1, HNF4α and HNF6 (Figure 5C and Figure 5D), indicating that GATA4 occupancy at these genes occurs only in hepatocytes and not in non-hepatic cell types. In conclusion, AAV-Cre-mediated excision allowed depletion of GATA4 from hepatocytes and eliminated its occupancy from target genes.

### Characterization of Gata4 excised adult livers

We evaluated the effects of deleting *Gata4* in hepatocytes on general mouse liver function. All AAV-Cre injected *Gata4 fl/fl* mice were viable and did not differ in weight from AAV-GFP injected mice (Figure S5A). We next examined serum markers indicative of liver function in mice with or without *Gata4* excision. Both albumin and urea are produced in the liver; hence their levels are indicative of the liver’s synthetic ability [57]. Levels of albumin and blood urea nitrogen (BUN), a byproduct of urea, were within normal range and did not differ between *Gata4* deleted and control hepatocytes (Figure S5B). Cholesterol and triglyceride levels also did not differ significantly between these groups of mice (Figure S5C). Alanine aminotransferase (ALT) and aspartate aminotransferase (AST), markers of liver injury, and alkaline phosphatase and total bilirubin, markers of hepatobiliary disease [58], were similar between control and *Gata4* excised mice (Figures S5D,E). Finally, we examined hematoxylin & eosin (H&E) stained liver sections for possible perturbations of the liver architecture in *Gata4* deficient hepatocytes. Livers from both groups of mice displayed normal tissue architecture, with intact central veins and portal tracts and no evidence of inflammation or fibrosis (Figure S5F-I). Thus, gross liver function as measured by serum markers and histology was not perturbed by deletion of GATA4 from hepatocytes.

Transcription factors can be largely dispensable under homeostatic conditions but might be required during tissue stress, such as that occurring following partial hepatectomy. However, tissue regeneration is not meant to recapitulate embryonic liver development, as distinct signaling pathways are required [59]. To address whether a requirement for GATA4 might be manifest under regenerative stress, we performed partial hepatectomies on *Gata4* excised and control mice. Liver weight at three days post-hepatectomy was used as measure of regrowth [59]. There was no significant difference in weights between *Gata4* excised and control groups (Figure S6), suggesting that liver regeneration does not require GATA4 expression. 

### Altered GATA4 target gene expression in Gata4 depleted hepatocytes

Since mice with *Gata4* deficient hepatocytes lacked gross phenotypic liver changes, we examined the effects of *Gata4* deletion on a transcriptional level. Hepatocytes were purified from both AAV-Cre and AAV-GFP injected *Gata4 fl/fl* mice. Depletion of endothelial and macrophage expressed genes confirmed purity of cell preparations (Figure S7). All cells or purified hepatocytes from both *Gata4* excised and control mice were examined by RT-qPCR for the expression of HNF4α, factor X, and albumin, which are normally hepatocyte-expressed genes (Figure 5E). None of these genes displayed altered expression levels upon *Gata4* excision. This was surprising since the factor X and HNF4α genes are both strongly bound by GATA4 at their promoters (Figure 5C and 5D) and factor X is a previously reported GATA4 target [30]. It is possible that GATA6 might occupy these genes and compensate for the loss of GATA4 but the lack of ChIP grade antibodies prevented us from performing anti-GATA6 ChIP experiments.

**Figure 6:**
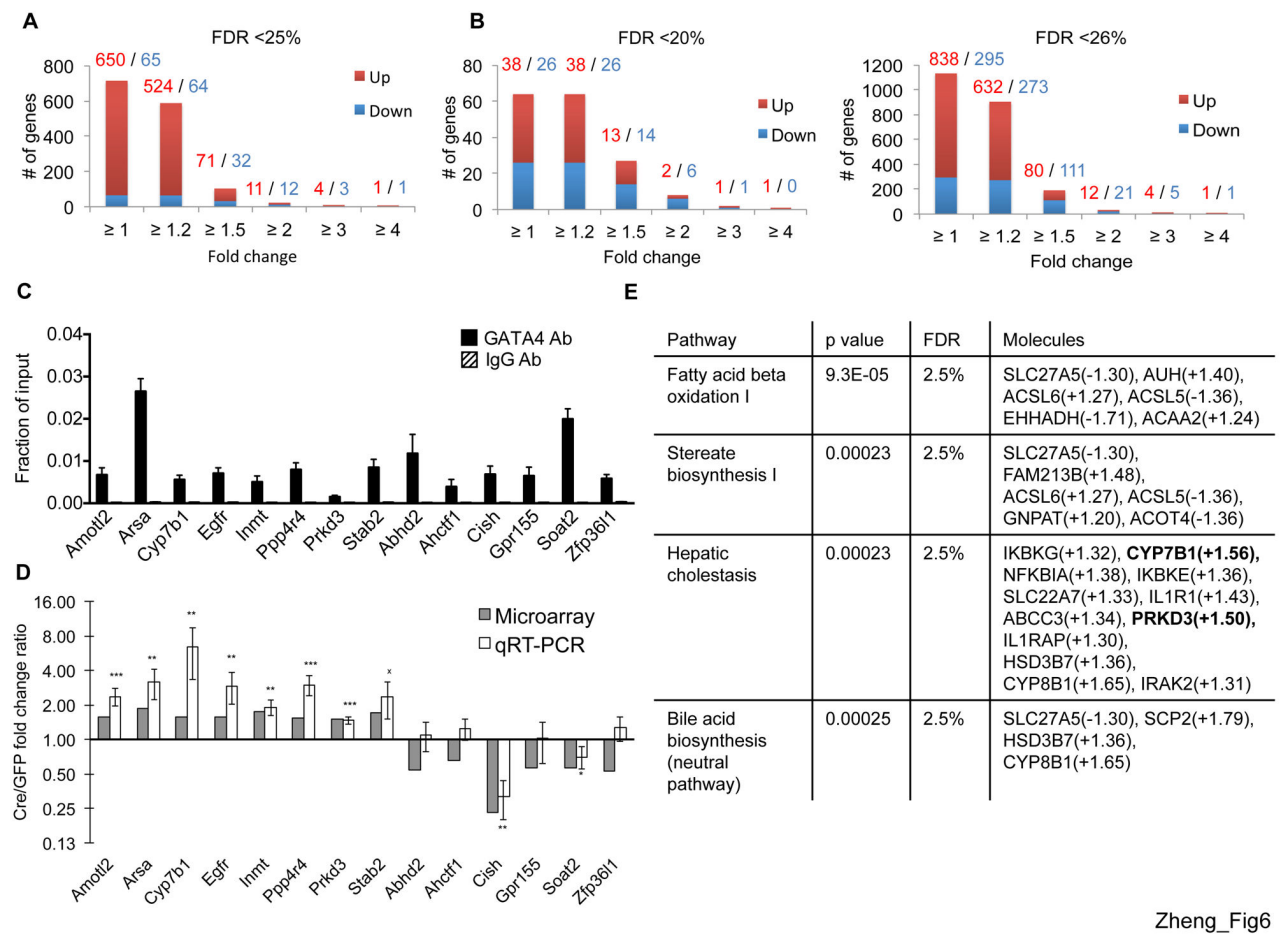
Transcriptomes of GATA4 excised and control hepatocytes. Number of differentially expressed genes at different fold change cutoffs with false discovery rate (FDR) threshold < 25% (A), <20% (B, left) and <26% (B, right). Red or blue numbers indicate upregulated and downregulated genes, respectively. (C) ChIP-qPCR shows GATA4 occupancy at differentially expressed genes. (D) Relative mRNA levels of differentially expressed genes. n=4, ***p<0.01, ** p<0.05, *p<0.07 by paired T test comparing fold change between AAV-Cre and AAV-GFP injected mice. (E) Ingenuity Pathway Analysis (IPA) shows enriched pathways of differentially expressed genes with FDR <25% and fold change >1.2. Note FDR and p-value on top row refer to values for specific pathways as determined by IPA. Bolded genes have at least one GATA4 OS within 5 kb upstream or downstream.

To identify on a global scale genes dependent on GATA4 in adult hepatocytes, we isolated mRNA from *Gata4* deleted and control hepatocytes obtained from mice two weeks after AAV injection. Transcripts were profiled using Illumina BeadChip microarrays. Significance of microarray (SAM) analysis identified 716 genes with a false discovery rate (FDR) less than 25%, without using fold-change cutoffs. Of these genes, 71 were upregulated and 32 were downregulated by at least 1.5 fold (Figure 6A). Initially, this finding was surprising because GATA factors are known to both activate and repress similar numbers of genes [50,60,61]. However, this imbalance was not observed when using either a more stringent FDR threshold of 20%, which yielded 13 upregulated and 14 downregulated genes or a more relaxed FDR threshold of 26%, revealing 80 upregulated and 111 downregulated genes, with both sets having at least a 1.5 fold change cut-off (Figure 6B). This highlights the importance of using multiple thresholds when assessing changes in gene expression patterns. Importantly, as shown below, the validation rate is very high even under these relaxed FDR thresholds. 

To determine if differentially expressed genes were direct GATA4 targets, we intersected the 4409 liver GATA4 ChIP-seq OS with the genes differentially expressed by at least 1.5 fold, with FDR less than or equal to 25%. Fifteen out of 71 up-regulated and 11 out of 32 down-regulated genes are bound by GATA4 in a window that is -5 kb upstream of the TSS and +5 kb downstream of the TTS. When the window is extended to 10 kb from the TSS and TTS, 19 up-regulated and 13 down-regulated genes are bound by GATA4 (Tables S4 and S5). Since GATA factors can function over distances greater than 10 kb, it is certainly possible that a higher number of direct GATA4 regulated genes would emerge if larger distances were considered. ChIP-qPCR confirmed GATA4 occupancy at 14/14 of examined genes within a 5 kb from TSS and TTS window (Figure 6C). Eight out of 14 differentially expressed genes, *Amotl2*, *Arsa*, *Cyp7b1*, *Egfr*, *Inmt*, *Pppr4r4*, *Prkd3*, and *Cish*, showed statistically significant changes (p<0.05) that were validated by RT-qPCR (Figure 6D). Two additional genes, *Stab2* and *Soat2*, showed the same trend of differential expression in RT-qPCR as in the microarray, although they did not meet the significance level. We then performed ingenuity pathway analysis (IPA) of all microarray genes with an FDR <25% or an FDR <26% and either fold change >1 or >1.2. We used these relaxed FDR and fold-change thresholds because IPA analysis requires a larger number of genes in order to organize pathways that are statistically significant. The four pathways common to all these analyses include fatty acid beta oxidation, stereate biosynthesis I, hepatic cholestasis, and bile acid biosynthesis (Figure 6E), all of which describe liver-specific functions. Together, these data show that a small number of GATA4 targets were dysregulated in GATA4 depleted hepatocytes and have liver-specific functions.

### Characterization of double Gata4 and Gata6 excised adult livers

**Figure 7:**
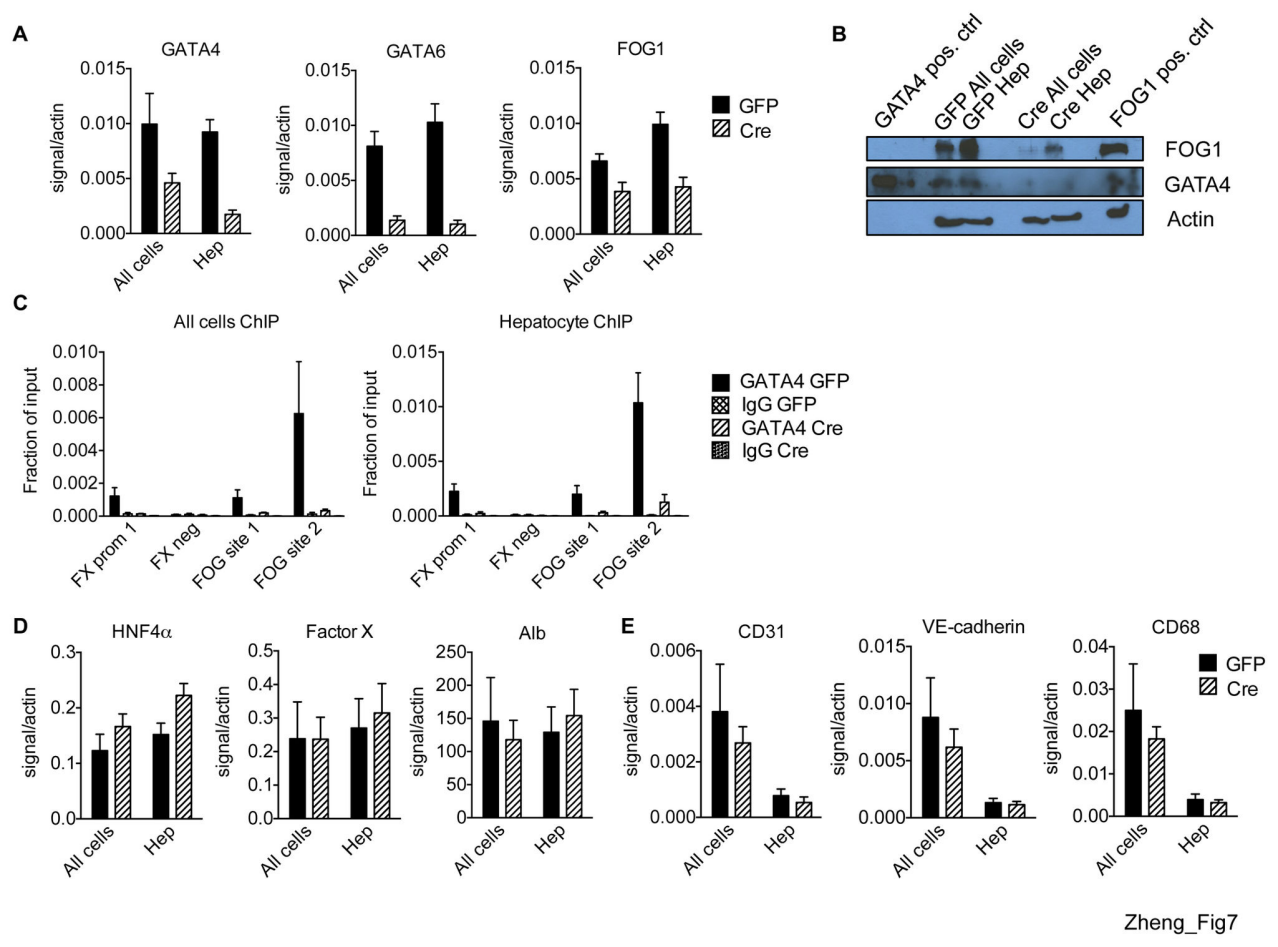
GATA4,6 double excision in hepatocytes. (A) mRNA levels of GATA4, GATA6, and FOG1 in all liver cells (all cells) or hepatocytes (hep) of AAV-GFP (black bars) and AAV-Cre (striped bars) injected mice (n=6). (B) Western blot for GATA4 and FOG1 proteins. Heart and mature erythroid cells served as positive controls (pos. ctrl) for GATA4 and FOG1, respectively. (C) GATA4 ChIP-qPCR in all cells or hep at factor X (FX) promoter (prom 1) and negative control region (neg) and FOG sites (n=2-4). mRNA levels of HNF4α, factor X, and albumin (D) and CD31, VE-cadherin, CD68 (E) in all cells or hep of AAV-GFP (black bars) or AAV-Cre (striped bars) injected mice (n=6).

 Since the mild phenotype observed in *Gata4* excised mice could be due to compensation by GATA6, we additionally excised *Gata6* from hepatocytes of adult mice. GATA4 and GATA6 mRNAs were depleted 2 weeks after excision (Figure 7A). FOG1 mRNA was reduced by two fold in *Gata4,6* double excised hepatocytes (Figure 7A) which is slightly greater than that seen in *Gata4* depleted hepatocytes, suggesting that GATA6 plays a role at activating FOG1. GATA4 protein was depleted and FOG1 protein was reduced in the *Gata4,6* double excised all cells and hepatocytes (Figure 7B). Importantly, GATA4 no longer occupied examined target genes in the *Gata4,6* double excised all cells and hepatocytes confirming virtual completeness of *Gata4* excision (Figure 7C).

 We next examined the phenotype of these mice. Excision of both *Gata4* and *Gata6* did not significantly affect body weight (Figure S8A). We measured the levels of the same serum markers that we had looked at previously for the *Gata4* single excision. Levels of serum BUN and albumin (Figure S8B), triglycerides and cholesterol (Figure S8C), ALT and AST (Figure S8D), and alkaline phosphatase and total bilirubin (Figure S8E) are not significantly different between *Gata4,6* double excised and control mice. H&E staining of liver sections from both groups of mice showed normal architecture, intact central veins and portal tracts, and no evidence of inflammation or fibrosis (Figure S8F-I). 

 We next examined the expression of hepatocyte-specific genes strongly occupied by GATA4 and possibly also by GATA6 in purified hepatocytes. Endothelial and macrophage genes were depleted in both control and excised hepatocytes, indicating proper enrichment of the hepatocyte fraction (Figure 7E). Unexpectedly, expression of none of these genes (HNF4α, factor X, and albumin) were changed in the doubly excised all cells populations or purified hepatocytes (Figure 7D). Other well characterized genes regulated by hepatocyte transcription factors include those of the serpin gene cluster [62]. Although GATA4 clearly occupied some of them, none change in expression following depletion of *Gata4* and *Gata6* (data not shown).

**Figure 8:**
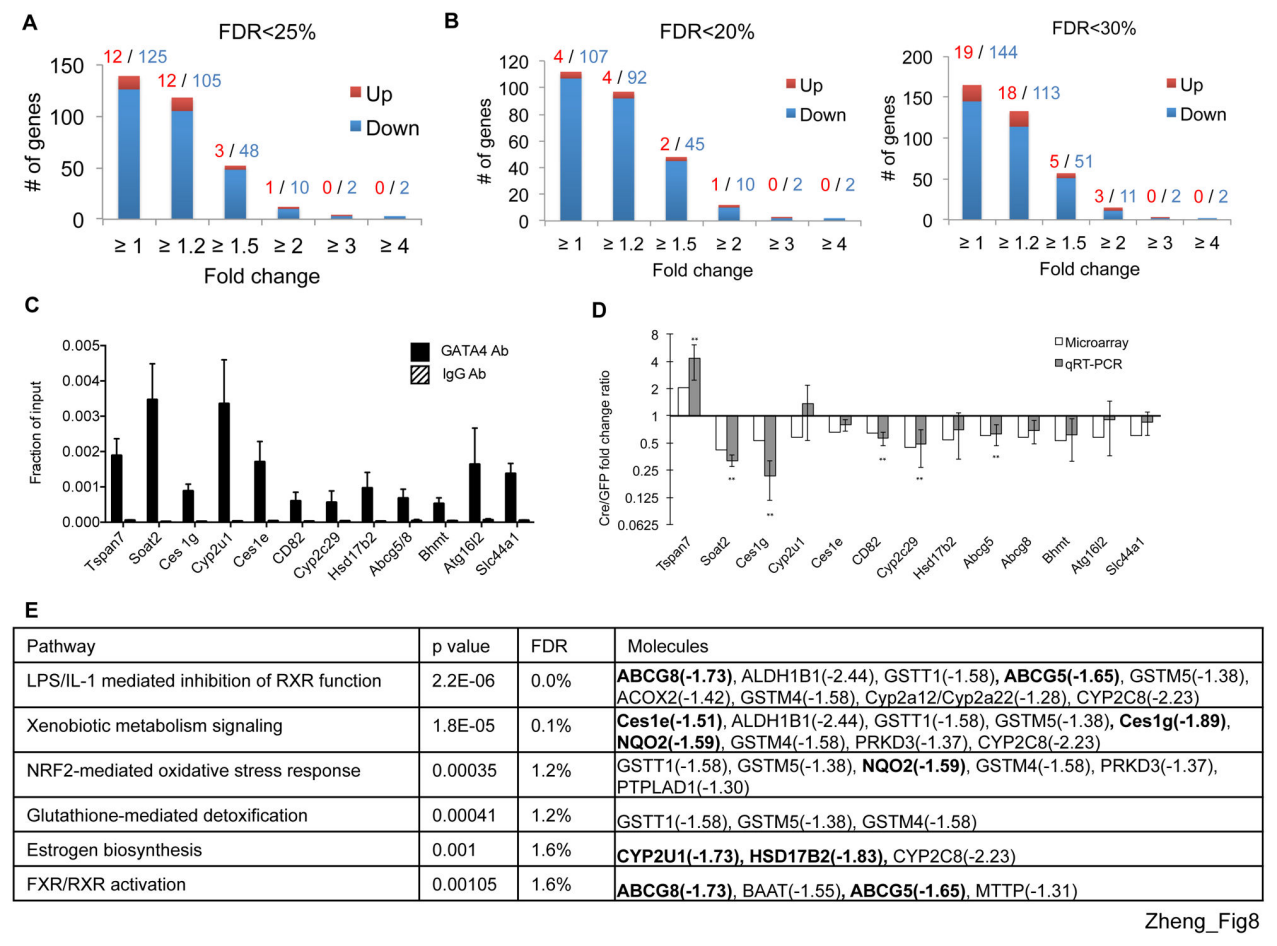
Transcriptomes of GATA4,6 double excised mice. Number of differentially expressed genes at different fold-change cutoffs with false discovery rate (FDR) thresholds < 25% (A), <20% (B, left) and <30% (B, right). Red or blue numbers indicate upregulated or downregulated genes, respectively. (C) ChIP-qPCR shows GATA4 occupancy at differentially expressed genes. (D) Relative mRNA levels of differentially expressed genes. n=4, ** p<0.05 by paired T test comparing fold change between AAV-Cre and AAV-GFP injected mice. (E) Ingenuity Pathway Analysis (IPA) shows enriched pathways of differentially expressed genes with FDR <30% and fold change >1.2. Note FDR and p-value on top row refer to values for specific pathways as determined by IPA. Bolded genes have at least one GATA4 OS within 10 kb upstream or downstream. FXR = farnesoid X receptor, RXR = retinoid X receptor.

To extend our gene expression analyses to a global scale, we examined the transcriptome of *Gata4,6* double excised hepatocytes and control hepatocytes (n=6) using Illumina BeadChip. SAM analysis identified 137 genes that met an FDR threshold of less than 25%, without any fold change cutoffs. At FDR<25% with a fold change greater than 1.5, there are 3 upregulated and 48 downregulated genes (Figure 8A). At a more relaxed threshold, FDR<30% with a fold change greater than 1.5, there are 5 upregulated and 51 downregulated genes. Hence, the number of downregulated exceed that of upregulated genes at all examined FDR thresholds and multiple fold change cut-offs (Figure 8A-C). This suggests that the majority of differentially expressed genes are potentially activated by GATA4 and/or GATA6. At first glance this is distinct from the more even distribution of upregulated and downregulated genes *Gata4* excised hepatocytes (Figure 6 and see discussion above). In addition, the total number of genes with FDR<25% and fold change>1.5 is almost two fold higher in *Gata4* excised than double *Gata4,6* excised hepatocytes. Unless GATA4 and GATA6 exert antagonistic functions on hepatic gene expression, it is hard to imagine that the combined GATA factor excision should produce less pronounced effects than single factor excision. Most likely, this apparent discrepancy is rooted in variation among the samples that were derived from outbred mice. Since the changes in gene expression levels are small in all samples and low fold-change thresholds were applied, small variations lead to an overrepresentation of differentially expressed genes. We next intersected the genes with FDR<30% and fold change >1.5 with the GATA4 ChIP-seq dataset. One upregulated and 22 downregulated genes had at least one GATA4 OS within 10 kb of the TSS and TTS (Table S6). A subset of these genes was validated by ChIP-qPCR (Figure 8C) and RT-qPCR (Figure 8D). Although all genes were occupied by GATA4, only approximately half of these displayed statistically significant changes in expression, including *Tspan7*, *Soat2*, *Ces1g*, *Cd82*, *Cyp2c29*, and *Abcg5*. The remaining half trended in the same direction as the microarray data, but a relatively high degree of variation among the samples reduced the statistical significance. We then performed ingenuity pathway analysis (IPA) of all microarray genes with an FDR <25% or an FDR <30% and fold change >1.2. Common ontologies to these analyses include farnesoid X receptor (FXR) and retinoid X receptor (RXR) function and detoxification related processes (Figure 8E). 

In summary, GATA4 and GATA6 are both expressed in hepatocytes and GATA4 can bind thousands of liver specific target genes. Yet, surprisingly, excision of *Gata4* alone or together with *Gata6* from hepatocytes led to minimal phenotypes. We are not aware of other cases in which the only two members of a transcription factor family expressed in a given tissue are both largely dispensable for function. This points to an unusually stable transcription program that is resistant to the perturbation of two transcription factors that in the context of other tissues and genes are potent activators of transcription. 

## Discussion

Here we investigated the role of GATA4 in adult livers by examining its expression pattern, genome-wide distribution, and function after hepatocyte-specific conditional deletion. GATA4 mRNA and protein are clearly detected in adult liver hepatocytes. ChIP followed by deep sequencing revealed that GATA4 occupies genes associated with liver specific functions. Hepatocyte specific conditional excision of *Gata4* led to complete loss of occupancy from its target genes, validating the specificity of the ChIP studies and pointing to the existence of a hepatocyte-specific gene expression program governed by GATA4. Although livers bearing GATA4 deficient hepatocytes appeared grossly and histologically normal, transcriptome analysis comparing wild type and excised hepatocytes combined with ChIP-seq data sets identified a small number of direct GATA4 target genes with liver specific functions. Surprisingly, additional deletion of *Gata6*, which was deemed capable of compensating for loss of *Gata4*, did not exacerbate the mild phenotype. Together, these results show that although GATA4 regulates liver specific transcription in adult hepatocytes, it is not essential for adult liver function.

Our observation that GATA4 is expressed in hepatocytes of the adult liver contrasts with IHC based reports that GATA4 is exclusively expressed in adult non-parenchymal cells, in particular endothelial cells and cholangiocytes [19-21,26]. Differences in methods measuring GATA4 expression as well as the low expression levels in hepatocytes might account for this discrepancy. Indeed, our own anti-GATA4 IHC studies of adult livers detected GATA4 predominantly in endothelial cells while the signal in hepatocytes was difficult to discern from background (not shown). Given the limitations in sensitivity of available GATA4 antibodies as well as the semi-quantitative nature of IHC, we took the approach of purifying hepatocytes and performing RT-qPCR and western blotting to measure GATA4 expression in these cells. Based on these experiments we estimate the amount of GATA4 in purified hepatocytes to be approximately half of that in all cells. Because hepatocytes account for ~70% of liver cells, less abundant cell types such as cholangiocytes and endothelial cells express substantially more GATA4 per cell than hepatocytes, thus explaining the observations with IHC.

To confirm and extend the expression studies and to identify the genomic locations of GATA4 we performed ChIP and ChIP-seq, which produced a robust set of 4409 GATA4 OS corresponding to 3075 genes. We chose whole livers for ChIP seq analysis because of the relative ease in obtaining sufficient quantities of tissue. The GATA4 occupancy pattern clearly favored hepatocyte-specific genes (see below). Nevertheless, it remained possible that this occupancy pattern was produced by GATA4 in non-hepatocytes. To address whether the GATA4 OS reflect a bone fide hepatocytic occupancy profile, and to establish that the anti-GATA4 antibodies are specific, we performed ChIP-qPCR in all cells and hepatocytes following hepatocyte specific GATA4 excision. In both samples, GATA4 was depleted from all targets examined, validating that GATA4 occupies its targets in a hepatocyte specific manner. 

Comparison of GATA4 OS data sets with those obtained from the heart [48] revealed that the majority of GATA4 OS are specific to the liver with only 20% overlapping with heart GATA4 OS. Notably, the liver selective GATA4 OS are bound to genes that have liver specific ontologies, including metabolism of lipids and lipoproteins, phase-1 functionalization of compounds, and glycolysis/gluconeogenesis, which are prominent hepatocyte specific functions [18]. Taken together, GATA4 is not only expressed in hepatocytes but also directly occupies hepatocyte-specific genes in these cells. 

To examine the function of GATA4 in the adult liver, we excised it specifically in hepatocytes using cre recombinase driven by a hepatocyte specific promoter in a viral vector with hepatocyte tropism. Mice lacking *Gata4* have outwardly normal livers with ostensibly normal histology, and exhibit levels of serum proteins similar to control mice. Nevertheless, using gene profiling we detected subtle changes in gene expression upon hepatocyte *Gata4* deletion, with 103 genes differentially expressed by at least 1.5 fold at an FDR threshold less than 25%. However, the number of differentially expressed genes after *Gata4* excision was only a small fraction of the total number of genes occupied by GATA4. This has also been observed in a cardiomyocyte cell line in which *Gata4* depletion affected only a small portion of GATA4 occupied genes [48]. Depending on whether a 5 kb or 10 kb window from the TSS or TTS is set, approximately 25-30% of differentially expressed genes are bound by liver GATA4. Again, this has been seen with GATA1, where only 16% of genes differentially expressed following GATA1 activation are directly bound by GATA1 [60]. It is possible that we are underestimating the true number of targets if GATA4 regulates some genes from more distal sites. For example, GATA1 has been shown to regulate target genes from distant genomic sites, such as the beta globin locus in erythroid cells [40]. Of course, there are likely secondary effects on gene expression resulting from the loss of GATA4. 

A possible reason for the mild hepatic phenotype in the absence of GATA4 is potential redundancy with GATA6. In the mouse pancreas, conditional deletion of either *Gata4* or *Gata6* does not perturb organ formation, whereas double knockout of *Gata4* and *Gata6* leads to pancreatic agenesis and death shortly after birth [63]. In the developing heart heterozygosity of either *Gata4* or *Gata6* deletion is compatible with relatively normal heart development but compound heterozygote animals die in utero due to a spectrum of cardiovascular deficiencies [64].

To address the possibility that GATA6 is compensating for the absence of GATA4, we performed double *Gata4* and *Gata6* excision in hepatocytes. Surprisingly, despite depletion of both GATA4 and GATA6 mRNAs and GATA4 protein and target gene occupancy, we detected no difference in weight or serum protein levels in these mice. Although there were some variations between *Gata4* and *Gata4,6* depleted cells, it is fair to conclude that the additional loss of *Gata6* did not augment gene expression changes found in the single *Gata4* knockout.

One interesting finding from the transcriptome of the *Gata4,6* excised hepatocytes is that there are disproportionately more downregulated than upregulated genes, which held true at all FDR thresholds examined with fold change >1.5 or 2 fold. Interestingly, shRNA knockdown of GATA4 in a cardiac cell line yielded a similar pattern of disproportionate number of downregulated genes (approximately 3 fold more than upregulated), indicating a more prominent role of GATA4 at activating genes [48]. 

Although GATA4 has previously been shown to activate certain liver-related genes in hepatoma cell lines [30-33], our transcriptome and ChIP-sequencing analyses suggest that GATA4 also functions as a repressor at a small number of genes, which is not surprising in light of other GATA factors capable of activating and repressing gene transcription [16,50,61]. GATA4 repressed genes also have liver specific functions, such as *Cyp7b1*, an enzyme in the alternative pathway of bile acid synthesis [65] and epidermal growth factor receptor (Egfr), involved in both proliferation and apoptosis in liver cell types, depending on context of ligand binding and coupling to additional receptors [66]. GATA4 repression of genes involved in liver function might serve to balance differentiation with proliferation. Genes expressed in immature hepatocytes might be turned off as late maturation genes are activated.

GATA4 also activates genes that play roles in the liver, including sterol-O-acyltransferase 2 (*Soat2*), encoding an enzyme which mediates esterification of cholesterol [67], and cytokine inducible SH2-containing protein (*Cish*), involved in the negative regulation of signal transducer and activator of transcription (STAT) signaling which regulates a wide variety of metabolic and inflammatory functions in the liver [68].

GATA4 and GATA6, together with HNF4α, have been shown in HepG2 cell lines to activate the ATP-binding cassette sterol transporters, *Abcg5* and *Abcg8*, which are oppositely oriented genes in close proximity to each other [69]. ChIP-seq and ChIP-qPCR identified GATA4 occupancy at an intergenic region between these two genes. In addition, both were downregulated in double *Gata4,6* excised hepatocytes, consistent with a role for direct activation by GATA4 and/or GATA6 *in vivo* in the liver. Murine knockout studies of *Abcg5* and *Abcg8* have shown that they function to excrete cholesterol into the bile, which is important for homeostasis of cholesterol and plant sterols[70]. Furthermore, a recent study found that the liver X receptor also binds to the intergenic region between these genes and cooperates with GATA factors and HNF4α to activate these genes [71]. It is possible that in the adult liver GATA4 modulates regulatory pathways that are dynamically controlled under varying metabolic conditions. For example, cholesterol and bile acid metabolism are intricately controlled by the often opposing actions of nuclear receptors farnesoid X receptor and liver X receptor [72]. GATA4 may be another transcription factor that adds to the regulation of these liver functions. A contribution by GATA4 and/or GATA6 to metabolism might be revealed under high fat diet or dietary restriction of mice. 

A likely possibility is that while GATA4 serves an essential function in early development [19], its requirement might be less stringent in the adult liver. Other hepatic transcription factors such as HNF4α, HNF1α, HNF1β, HNF3β/Foxa2, and HNF6 form an increasingly robust cross-regulatory network during development from the embryonic to adult liver [73]. Deletion of *Hnf4α* in fetal livers affects the expression of other factors in the network to a greater degree than when it is depleted in adult liver, indicating that an already established network in mature livers can withstand the loss of individual factors without severe perturbation [73]. Embryonic livers with *Hnf4α* conditional deletion display severe loss of cell contacts between hepatocytes as well as defects in glucose metabolism [74] while mice lacking adult liver HNF4α display a much less severe phenotype, with reduced triglyceride and cholesterol levels and decreased expression of lipid metabolism and transport genes [75]. Another example is the role of HNF3β/FOXA2 in liver development. Lack of HNF3β/FOXA2 leads to reduced expression of critical liver regulators, including HNF3α, HNF1α and HNF4α, and decreased apolipoprotein and albumin expression in an in vitro differentiation assay for the earliest stages of liver development [76]. However, conditional *Hnf3β/Foxa2* deletion in late fetal development has minimal impact on adult liver morphology or gene expression suggesting that other factors can compensate for its loss [77]. Hence, adult liver transcription programs seem less sensitive to perturbations that those controlling early liver development.

It appears that in the homeostatic adult liver, both GATA4 and GATA6 are relatively dispensable for normal function. However, GATA4 and GATA6 function might only revealed during stress. For example, deletion of transcription factor forkhead box o3 (*Foxo3*) only mildly affects erythroid cell function, but strongly exacerbates anemia and hemolysis to the point of lethality upon oxidative stress [78]. Mice lacking the liver expressed nuclear orphan receptor pregnane X receptor (Pxr) display normal viability and normal serum protein and lipid levels. However, upon exposure to toxic bile acid, *Pxr* knockout mice develop more severe liver damage than wild type controls [79]. To examine whether a requirement for GATA4 is exposed under stress to the liver, we carried out partial hepatectomies in mice with hepatocyte specific *Gata4* depletion and found little difference in their regenerative potential when compared to controls. It will be important to examine whether *Gata4,6* doubly excised mice display defects when challenged with partial hepatectomy.

Preliminary observations show that mice with a mutation that disrupts the FOG1 and NuRD interaction exhibit a statistically significantly increased incidence of HCC compared to controls when aged beyond one to one and a half years. This finding implicates GATA4 and/or GATA6 as a potential tumor suppressor, since FOG1, unable to bind DNA, functions exclusively through GATA factors [1]. Exposure to hepatic carcinogens accelerated HCC formation in all animals but did not produce the same predilection for mutant mice that was observed in untreated mutant animals (not shown). Therefore, although tantalizing, a role of the GATA4/FOG1/NuRD axis as potential tumor suppressor remains unresolved at the present time requiring increased cohorts of aged animals. Additionally, it will be interesting to assess whether *Gata4* deleted mice develop HCC at an increased rate upon aging.

In conclusion, GATA4 is expressed in hepatocytes where it occupies a large set of liver specific genes and contributes to the expression of a subset of these genes. The mild consequences of *Gata4* and *Gata6* deletion on transcription and liver function suggest the existence of a robust network of regulatory factors with high compensatory potential in adult hepatocytes.

## Supporting Information

Figure S1 **Liver tumors in aged Fog1 knock-in mice.** (A) Table showing the fraction and frequency of wild type (WT), Fog1 heterozygous (ki/+) and Fog1 homozygous (ki/ki) mice with macroscopic liver tumors, *p<0.05 by Fisher’s exact test. (B) Image of livers from WT and *Fog1* *ki/ki* mice. Note the multifocal tumors present in the ki/ki liver. (C) H&E staining of a *Fog1* *ki/ki* tumor section at 20X magnification identifies it as hepatocellular carcinoma. Tumor cells contain nuclear polymorphisms with hyperchromasia and chromatin clumping (a), prominent nucleoli (b), and mitotic Figures (c). Scale bar represents 100 μm.
(TIF)

Figure S2 **Validation of GATA4 OS.** (A) ChIP-qPCR of GATA4 OS with a range of IDR significance levels. (B) Genome browser screenshot showing prominent GATA4 ChIP-seq peak at factor X promoter (denoted by red bar). Factor X gene is marked with blue box. Y axis shows read counts for GATA4 ChIP-seq (upper track) and input DNA (lower track).
(TIF)

Figure S3 **Patterns of GATA4 occupied sites.** (A) Distribution of GATA4 occupied sites among gene elements with extended window, including 10 kb upstream of TSS to end of first exon (proximal promoter) and last exon to 10 kb downstream of TTS (see Figure 3 for legend of other elements). (B) Distribution of GATA4 occupied sites in regular 5 kb window (as shown in Figure 3) of unique to heart ChIP-seq dataset, excluding heart/liver common occupied sites. Intersection of combined H3K4me1 and H3K27ac occupied sites (enhancer marks) and intergenic GATA4 occupied sites outside of 5 kb (C) or 10 kb (D) windows.
(TIF)

Figure S4 **Non-canonical GATA motifs.** Fraction and percentage of peaks containing non-canonical GATA motifs WGATA or (A/T)GATA and GATAR or GATA(A/G) as identified by MEME motif analysis.
(TIF)

Figure S5 **Phenotype of mice with hepatocyte-specific deletion of *Gata4*.** (A) Fractional total body weight changes at one and two weeks after injection with AAV-GFP (circle) and AAV-Cre (triangle) (n=5-12). (B) Serum blood urea nitrogen (BUN) and albumin, (C) cholesterol and triglycerides, (D) alanine aminotransferase (ALT) and aspartate aminotransferase (AST), (E) alkaline phosphatase (alk phos) and total bilirubin in AAV-GFP and AAV-Cre injected mice (n=3-4). Reference ranges: BUN 18-29 mg/dL, albumin 2.5-4.8 g/dL, cholesterol 36-96 mg/dL, triglycerides 55-144 mg/dL, ALT 28-132 U/L, AST 59-347 U/L, alkaline phosphatase 52-209 U/L, total bilirubin 0.1-0.9 mg/dL. Hematoxylin and eosin (H&E) staining of liver section from AAV-GFP injected mice at 10X (F) and 20X (G) magnification and AAV-Cre injected mice at 10X (H) and 20X (I) magnification show intact tissue architecture, no fibrosis and no inflammation. Arrows point to central veins and arrowheads indicate portal tracts. Scale bars represent 100 μm.
(TIF)

Figure S6 **Liver weights after partial hepatectomy.** The liver weight to body weight ratio in *Gata4* *fl/fl* mice injected with AAV-Cre or AAV-GFP at 3 days after partial hepatectomy (phx) or sham surgery (sham).
(TIF)

Figure S7 **Enrichment of hepatocytes following purification from AAV-Cre and AAV-GFP injected *Gata4fl/fl* mice.** Relative mRNA levels as measured by RT-qPCR of endothelial cell markers CD31 and VE-cadherin, and Kupffer cell marker CD68 from all liver cells (all cells) or hepatocytes (hep) (n=5-7) of AAV-GFP (black bar) and AAV-Cre (striped bar) injected mice. Student’s unpaired t test was not significant when comparing all liver cells or hepatocytes from AAV-GFP and AAV-Cre injected mice.
(TIF)

Figure S8 **Phenotype of mice with hepatocyte specific deletion of *Gata4* and *Gata6*.** A) Fractional total body weight changes at one or two weeks after injection with AAV-GFP (circle) and AAV-Cre (triangle) (n=6-9). Student’s unpaired t test was not significant comparing weights for two groups. (B) Serum blood urea nitrogen (BUN) and albumin, (C) cholesterol and triglycerides, (D) alanine aminotransferase (ALT) and aspartate aminotransferase (AST), (E) alkaline phosphatase (alk phos) and total bilirubin in AAV-GFP and AAV-Cre injected mice (n=2). Student’s unpaired t test was not significant comparing any of the serum values between two groups. Reference ranges: BUN 18-29 mg/dL, albumin 2.5-4.8 g/dL, cholesterol 36-96 mg/dL, triglycerides 55-144 mg/dL, ALT 28-132 U/L, AST 59-347 U/L, alkaline phosphatase 52-209 U/L, total bilirubin 0.1-0.9 mg/dL. Note that albumin in Cre samples in panel B and total bilirubin in panel E have no error bars because the values were the same for both replicates. Hematoxylin and eosin (H&E) staining of liver section from AAV-GFP injected mice at 10X (F) and 20X (G) magnification and AAV-Cre injected mice at 10X (H) and 20X (I) magnification show intact tissue architecture, no fibrosis, and no inflammation. Arrows point to central veins and arrowheads indicate portal tracts. Scale bars represent 100 μm.
(TIF)

Table S1 **Genotyping primer sequences.** Sequences for forward and reverse genotyping primers for each mouse strain.
(PDF)

Table S2 **RT-qPCR primer sequences.** Sequences for forward and reverse RT-qPCR primers.
(PDF)

Table S3 **ChIP primer sequences.** Sequences for forward and reverse ChIP-qPCR primers.
(PDF)

Table S4 **Genes up-regulated upon *Gata4* excision that contain at least one GATA4 OS.** “Start” and “end” denote genomic coordinates of GATA4 OS. (chr=chromosome, plus sign (+) represents upregulated). Note that some genes have more than one GATA4 OS.
(PDF)

Table S5 **Genes down-regulated upon *Gata4* excision that contain at least one GATA4 OS.** “Start” and “end” denote genomic coordinates of GATA4 OS. (chr=chromosome, negative sign (-) represents downregulated). Note that some genes have more than one GATA4 OS.
(PDF)

Table S6 **Differentially expressed genes upon *Gata4,6* double excision that contain at least one GATA4 OS.** “Start” and “end” denote genomic coordinates of GATA4 OS. (chr=chromosome, positive sign (+) or negative sign (-) represent upregulated or downregulated, respectively). Note that some genes have more than one GATA4 OS.
(PDF)
