# Supplementary figures and images for: Function of GATA Factors in the Adult Mouse Liver
Source: PLoS One. 2013 Dec 18;8(12):e83723. doi: 10.1371/journal.pone.0083723 (PMC3867416; doi:10.1371/journal.pone.0083723)

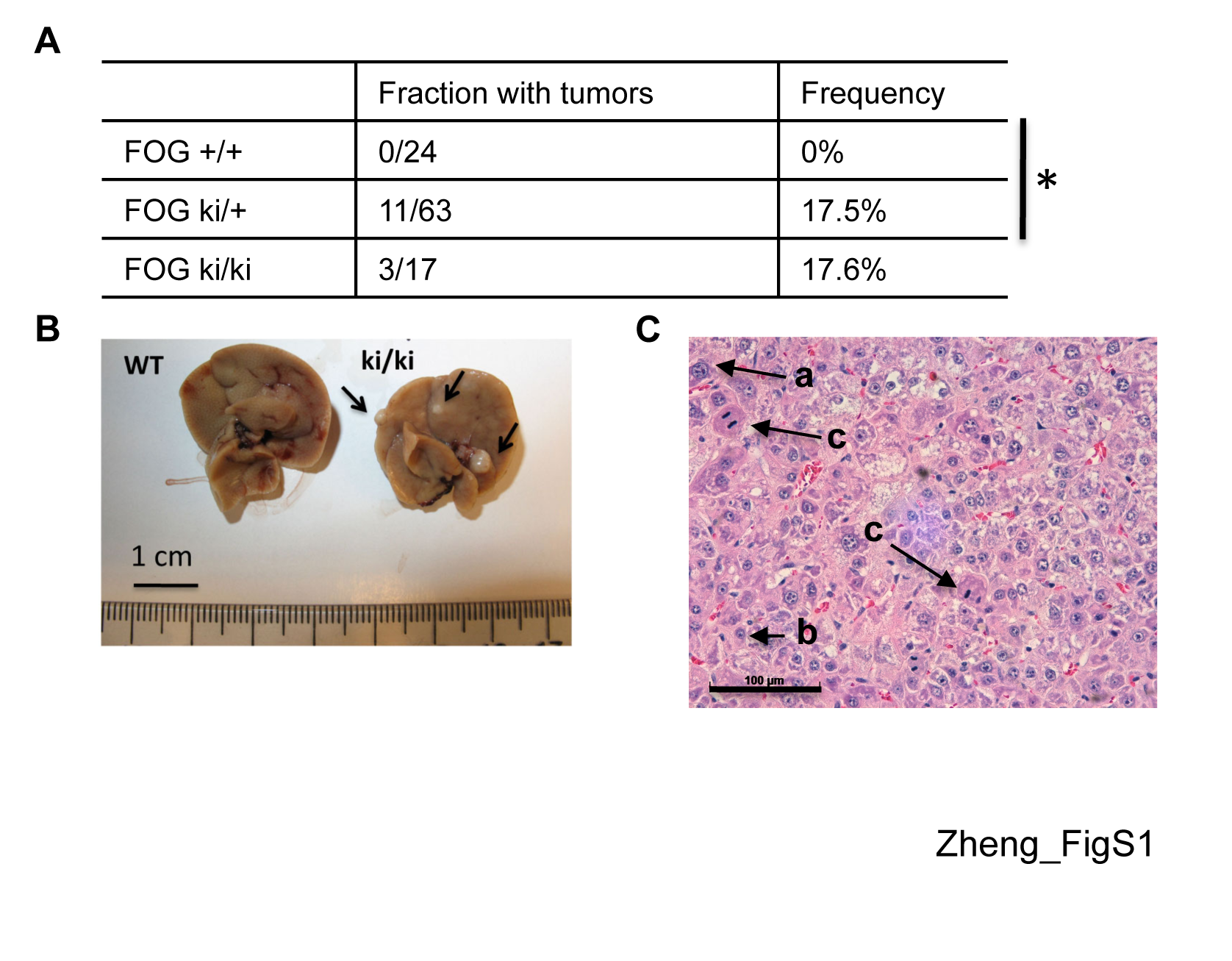

Supplement: Figure S1 — Liver tumors in aged Fog1 knock-in mice. (A) Table showing the fraction and frequency of wild type (WT), Fog1 heterozygous (ki/+) and Fog1 homozygous (ki/ki) mice with macroscopic liver tumors, *p<0.05 by Fisher’s exact test. (B) Image of livers from WT and Fog1 ki/ki mice. Note the multifocal tumors present in the ki/ki liver. (C) H&E staining of a Fog1 ki/ki tumor section at 20X magnification identifies it as hepatocellular carcinoma. Tumor cells contain nuclear polymorphisms with hyperchromasia and chromatin clumping (a), prominent nucleoli (b), and mitotic Figures (c). Scale bar represents 100 μm. (TIF) [file pone.0083723.s001.tif]

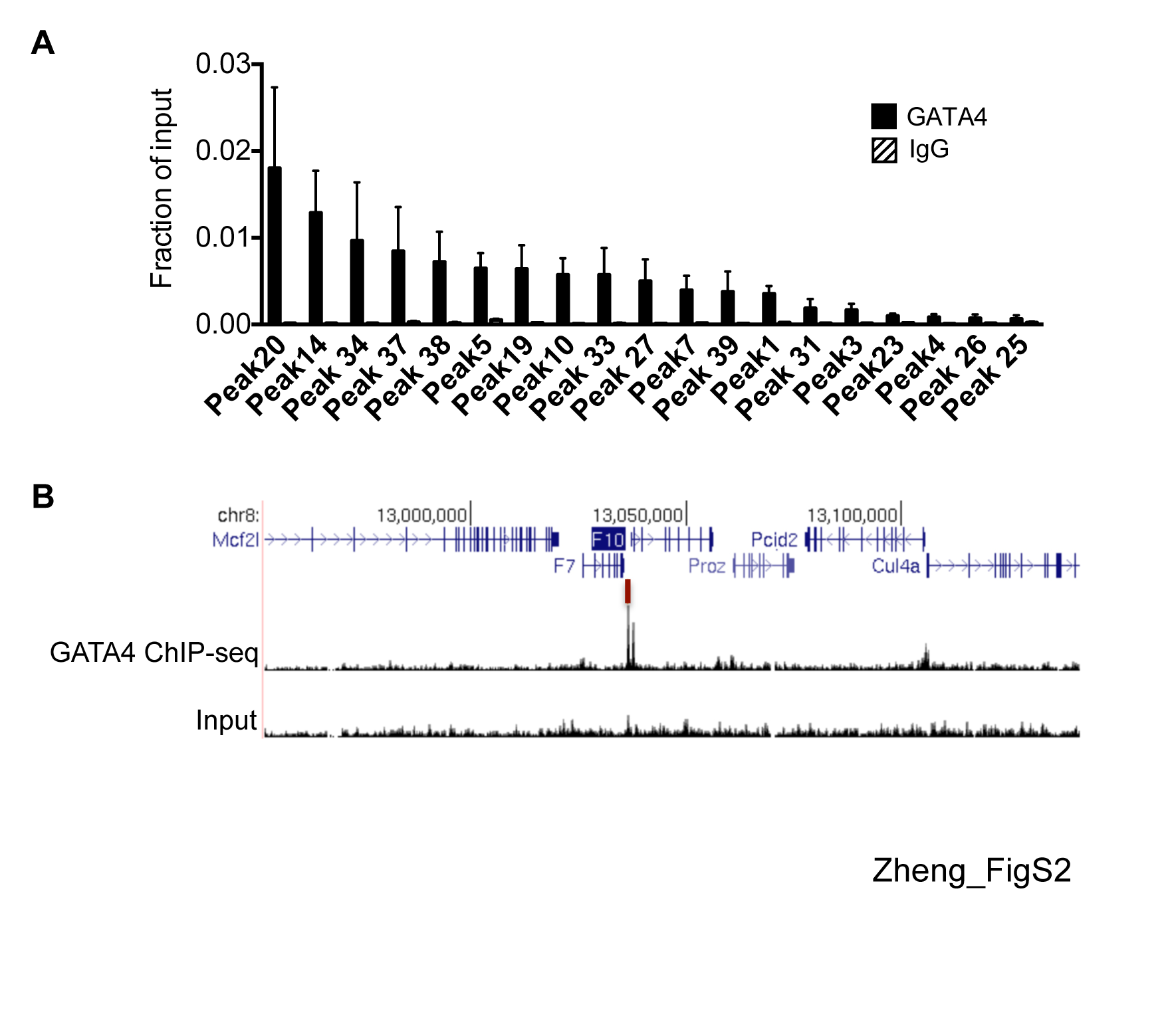

Supplement: Figure S2 — Validation of GATA4 OS. (A) ChIP-qPCR of GATA4 OS with a range of IDR significance levels. (B) Genome browser screenshot showing prominent GATA4 ChIP-seq peak at factor X promoter (denoted by red bar). Factor X gene is marked with blue box. Y axis shows read counts for GATA4 ChIP-seq (upper track) and input DNA (lower track). (TIF) [file pone.0083723.s002.tif]

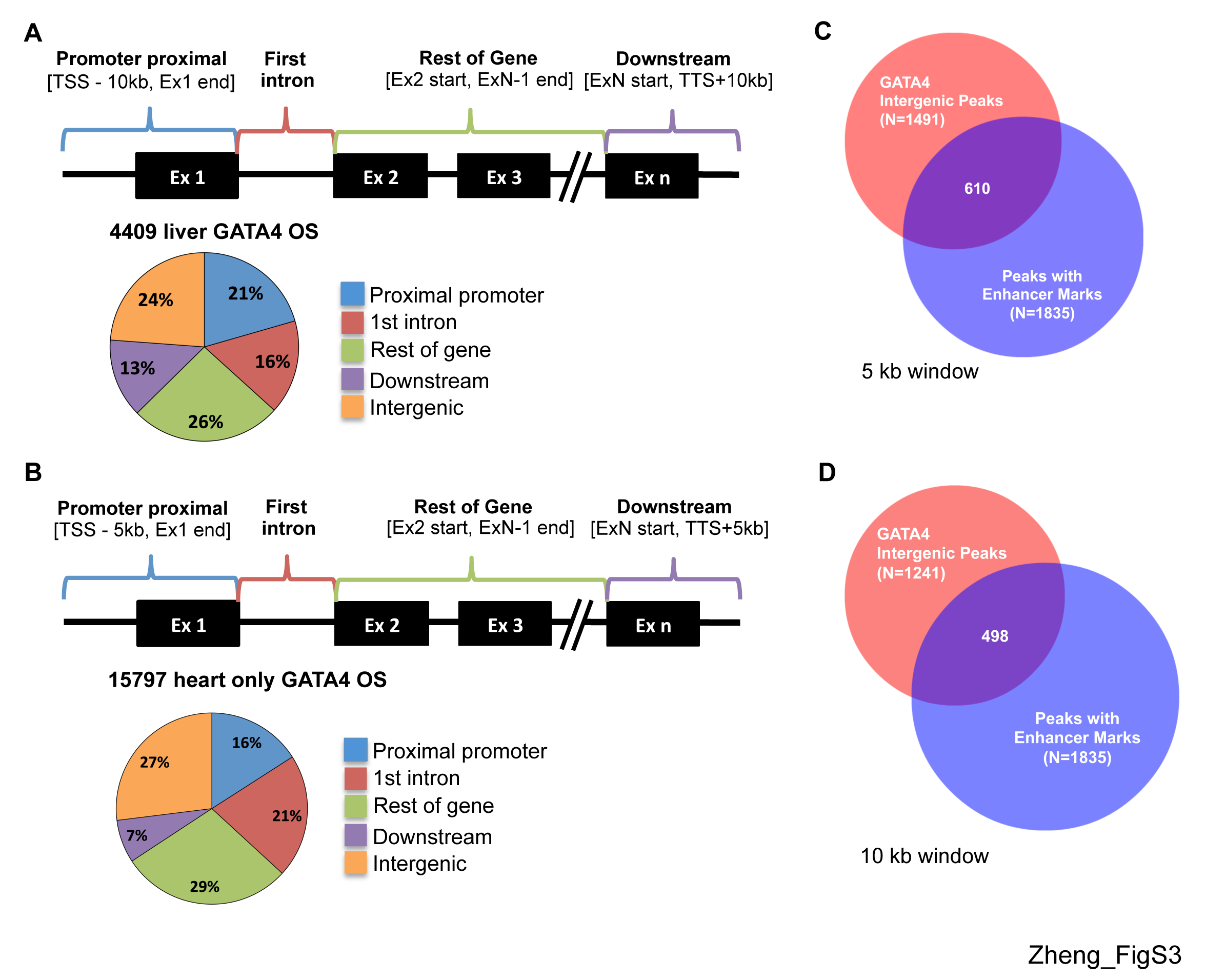

Supplement: Figure S3 — Patterns of GATA4 occupied sites. (A) Distribution of GATA4 occupied sites among gene elements with extended window, including 10 kb upstream of TSS to end of first exon (proximal promoter) and last exon to 10 kb downstream of TTS (see Figure 3 for legend of other elements). (B) Distribution of GATA4 occupied sites in regular 5 kb window (as shown in Figure 3) of unique to heart ChIP-seq dataset, excluding heart/liver common occupied sites. Intersection of combined H3K4me1 and H3K27ac occupied sites (enhancer marks) and intergenic GATA4 occupied sites outside of 5 kb (C) or 10 kb (D) windows. (TIF) [file pone.0083723.s003.tif]

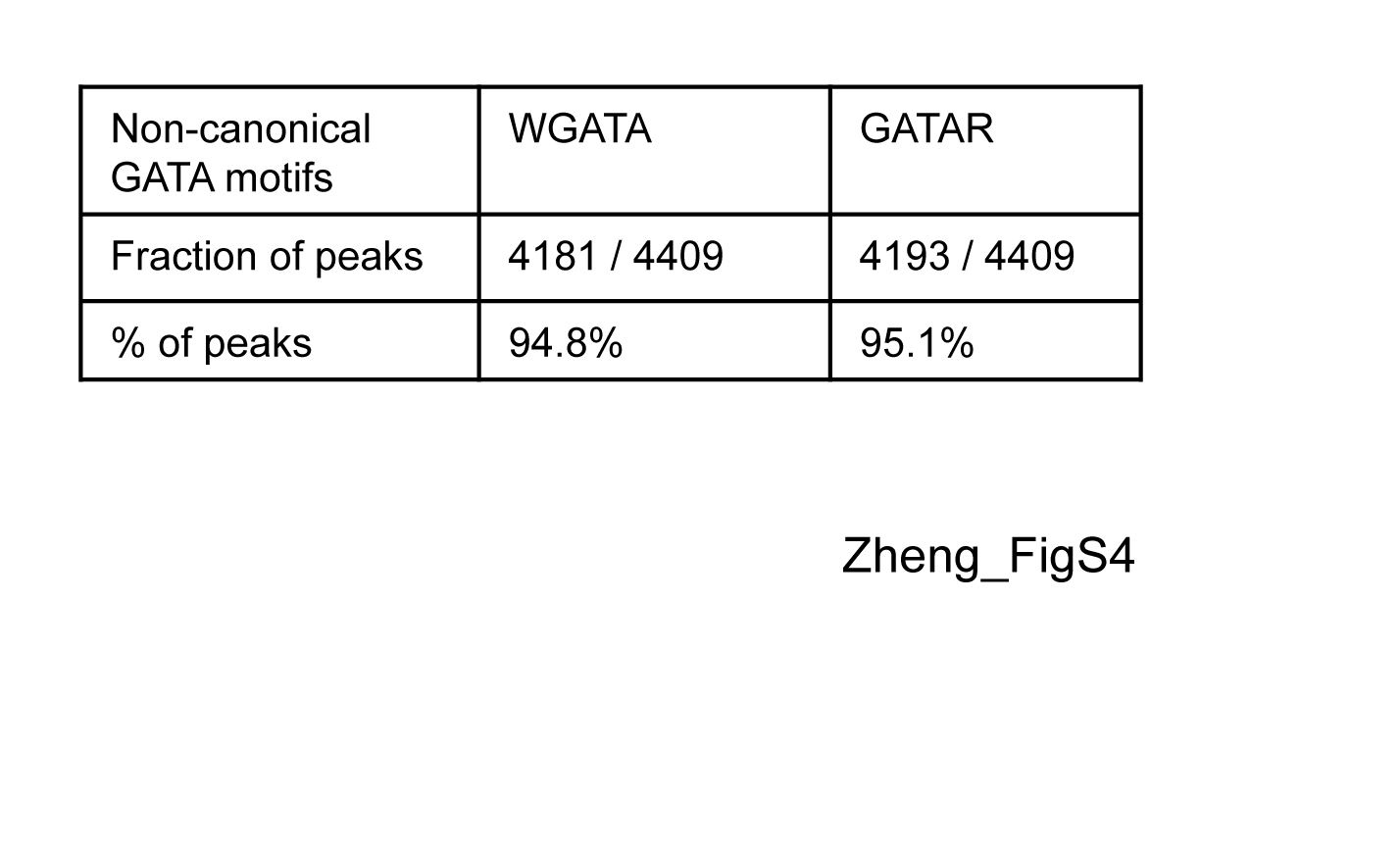

Supplement: Figure S4 — Non-canonical GATA motifs. Fraction and percentage of peaks containing non-canonical GATA motifs WGATA or (A/T)GATA and GATAR or GATA(A/G) as identified by MEME motif analysis. (TIF) [file pone.0083723.s004.tif]

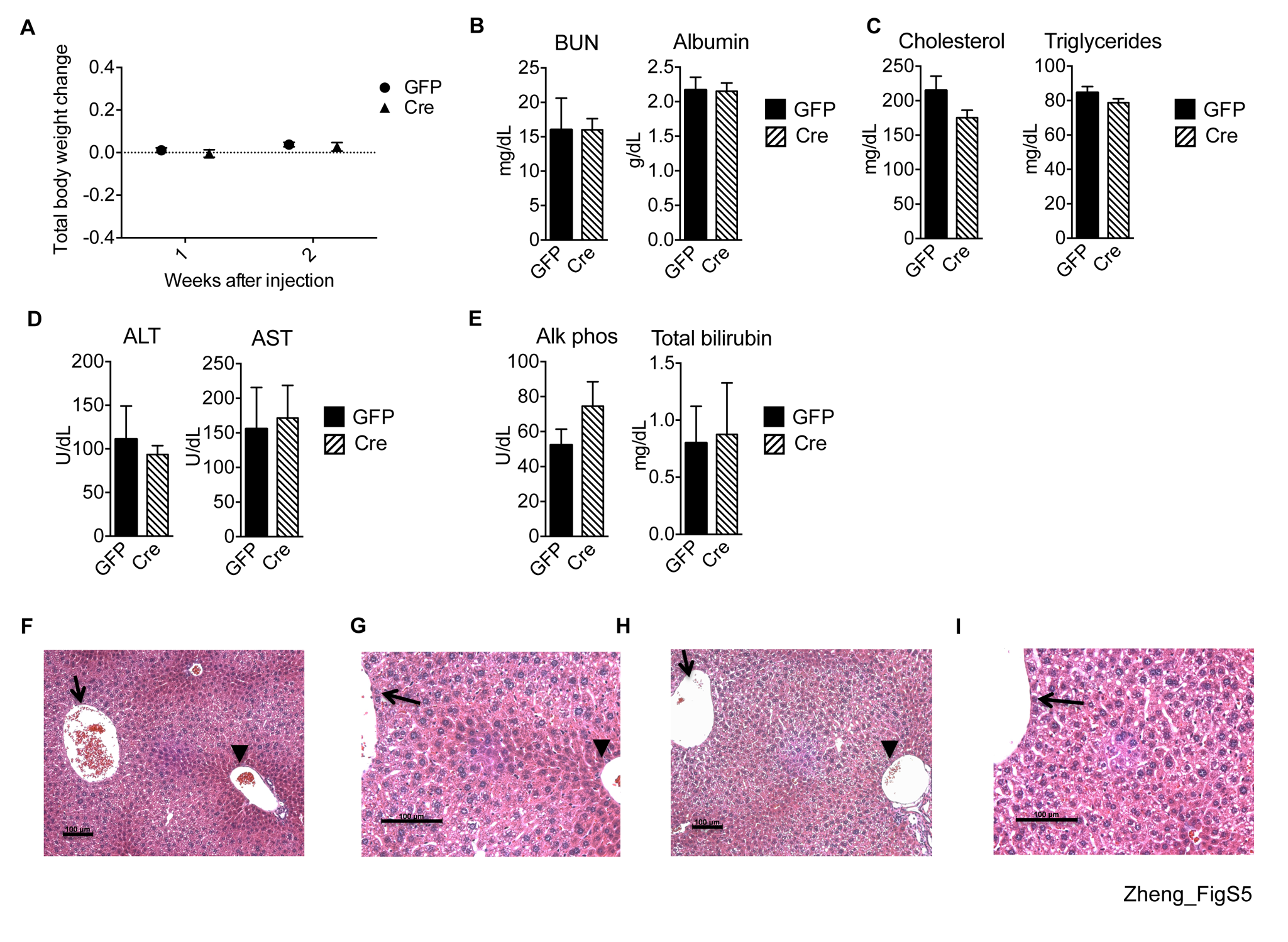

Supplement: Figure S5 — Phenotype of mice with hepatocyte-specific deletion of Gata4. (A) Fractional total body weight changes at one and two weeks after injection with AAV-GFP (circle) and AAV-Cre (triangle) (n=5-12). (B) Serum blood urea nitrogen (BUN) and albumin, (C) cholesterol and triglycerides, (D) alanine aminotransferase (ALT) and aspartate aminotransferase (AST), (E) alkaline phosphatase (alk phos) and total bilirubin in AAV-GFP and AAV-Cre injected mice (n=3-4). Reference ranges: BUN 18-29 mg/dL, albumin 2.5-4.8 g/dL, cholesterol 36-96 mg/dL, triglycerides 55-144 mg/dL, ALT 28-132 U/L, AST 59-347 U/L, alkaline phosphatase 52-209 U/L, total bilirubin 0.1-0.9 mg/dL. Hematoxylin and eosin (H&E) staining of liver section from AAV-GFP injected mice at 10X (F) and 20X (G) magnification and AAV-Cre injected mice at 10X (H) and 20X (I) magnification show intact tissue architecture, no fibrosis and no inflammation. Arrows point to central veins and arrowheads indicate portal tracts. Scale bars represent 100 μm. (TIF) [file pone.0083723.s005.tif]

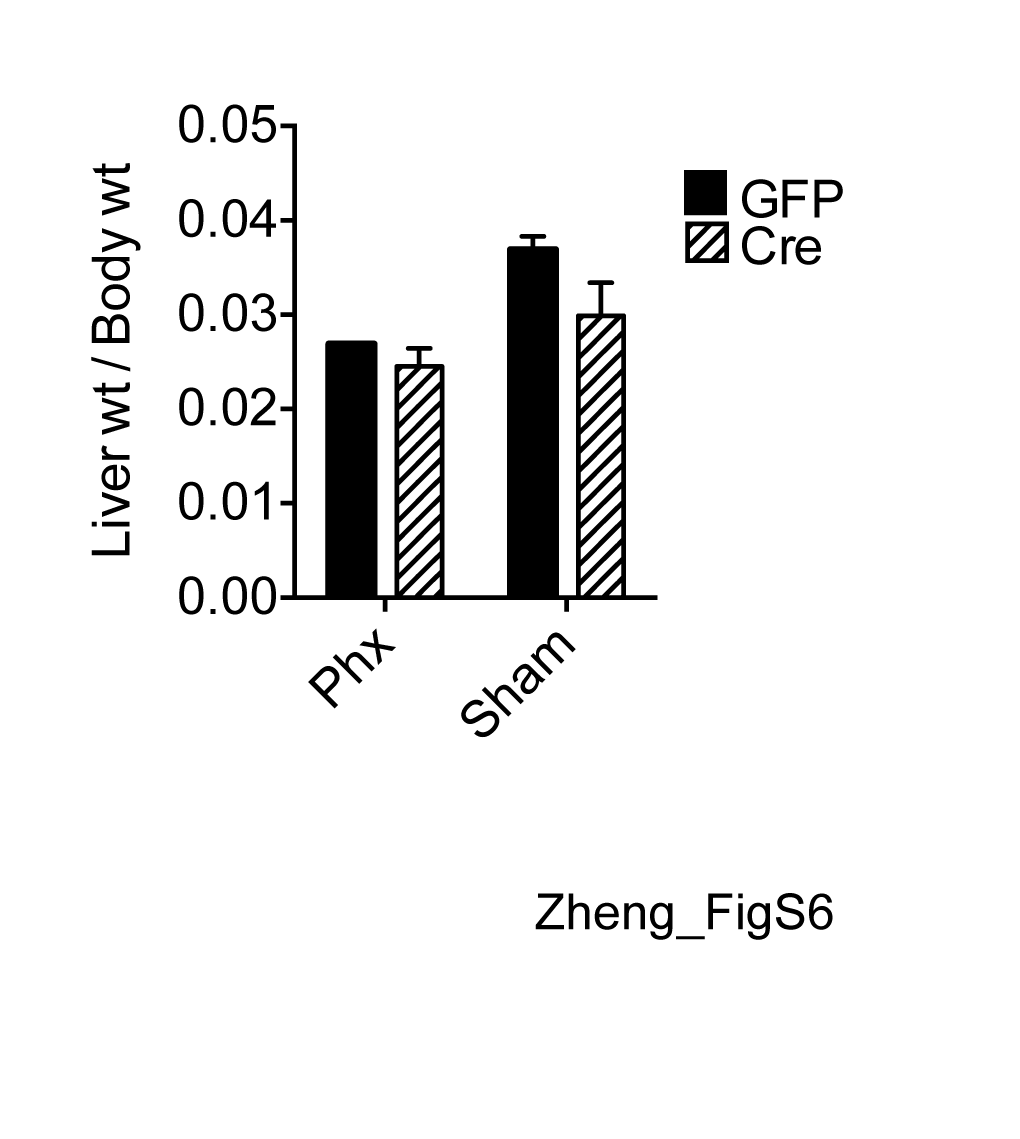

Supplement: Figure S6 — Liver weights after partial hepatectomy. The liver weight to body weight ratio in Gata4 fl/fl mice injected with AAV-Cre or AAV-GFP at 3 days after partial hepatectomy (phx) or sham surgery (sham). (TIF) [file pone.0083723.s006.tif]

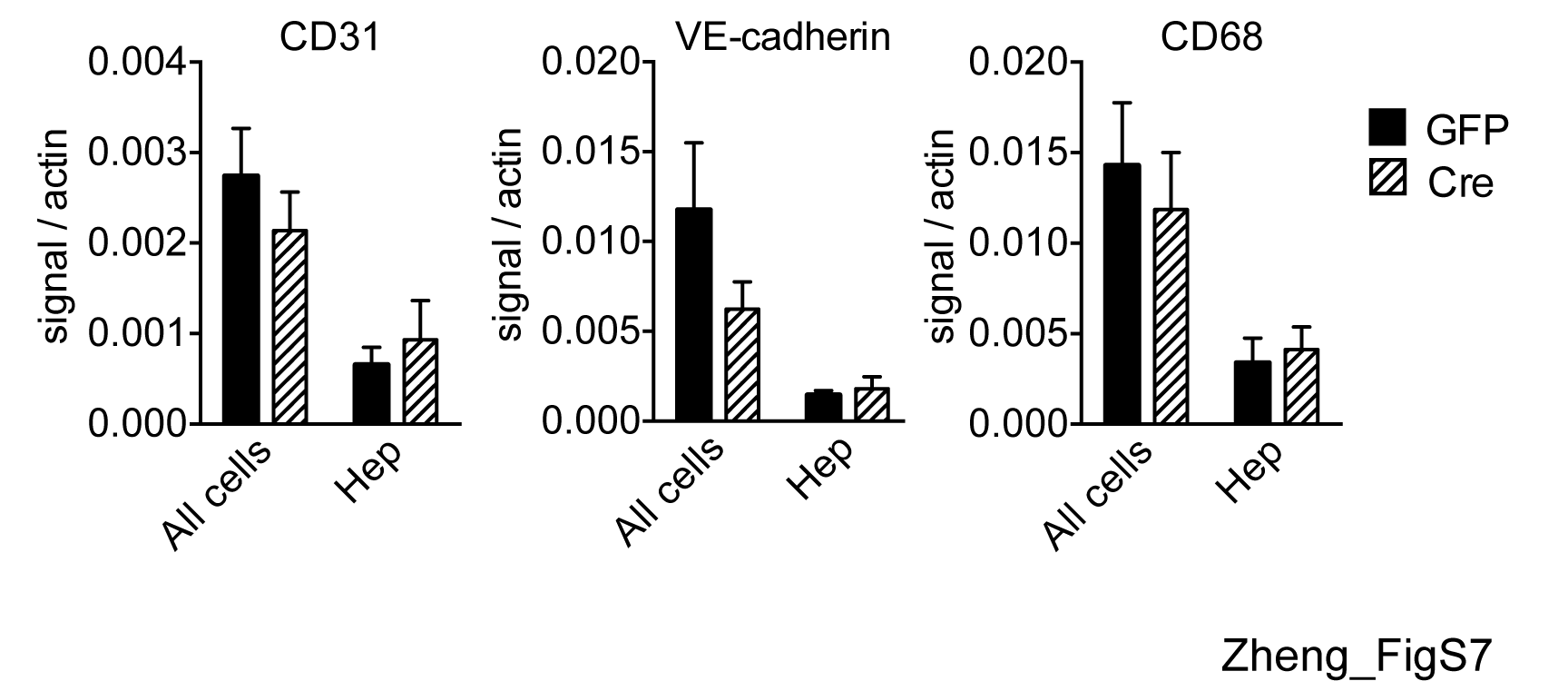

Supplement: Figure S7 — Enrichment of hepatocytes following purification from AAV-Cre and AAV-GFP injected Gata4fl/fl mice. Relative mRNA levels as measured by RT-qPCR of endothelial cell markers CD31 and VE-cadherin, and Kupffer cell marker CD68 from all liver cells (all cells) or hepatocytes (hep) (n=5-7) of AAV-GFP (black bar) and AAV-Cre (striped bar) injected mice. Student’s unpaired t test was not significant when comparing all liver cells or hepatocytes from AAV-GFP and AAV-Cre injected mice. (TIF) [file pone.0083723.s007.tif]

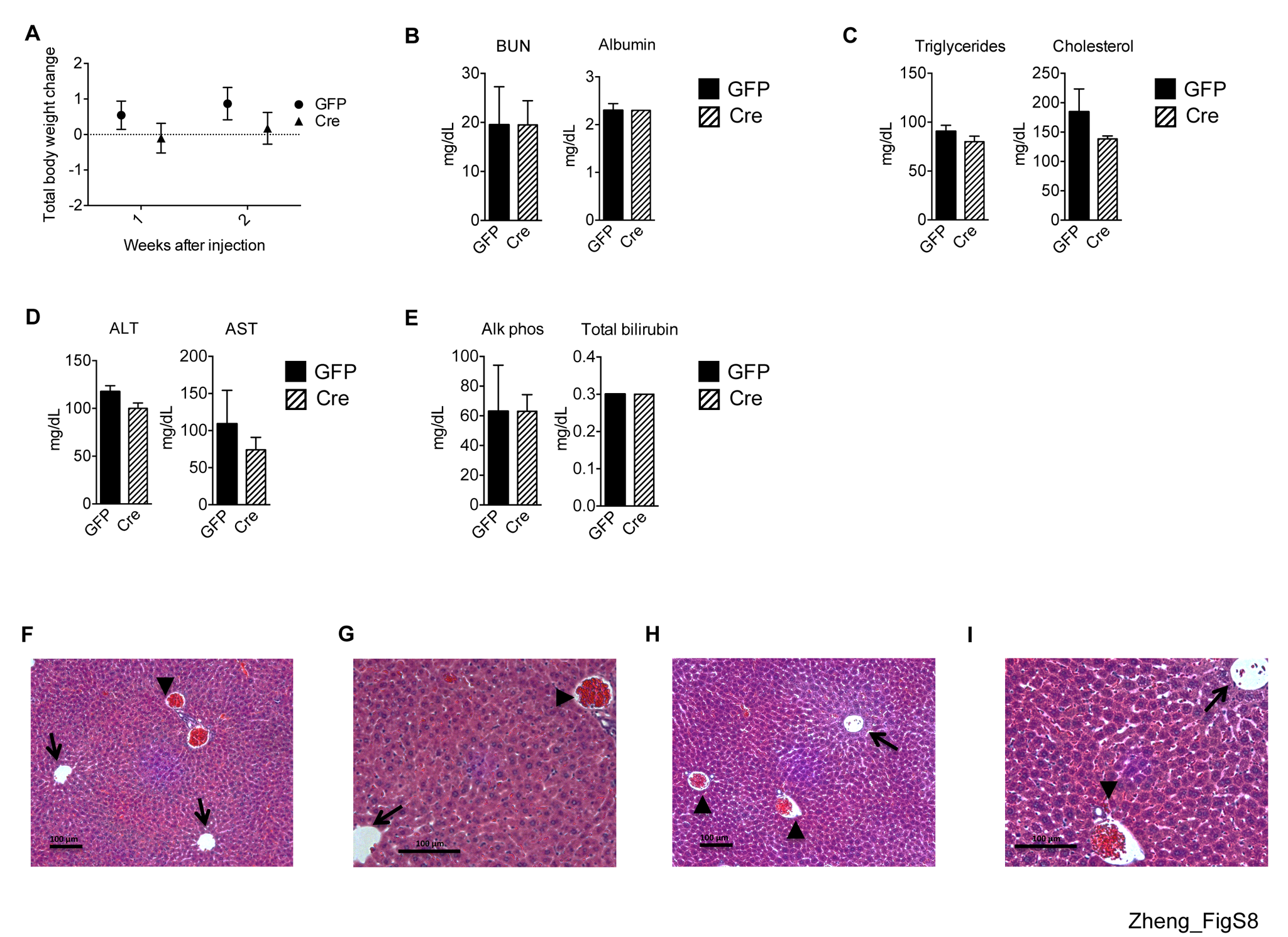

Supplement: Figure S8 — Phenotype of mice with hepatocyte specific deletion of Gata4 and Gata6. A) Fractional total body weight changes at one or two weeks after injection with AAV-GFP (circle) and AAV-Cre (triangle) (n=6-9). Student’s unpaired t test was not significant comparing weights for two groups. (B) Serum blood urea nitrogen (BUN) and albumin, (C) cholesterol and triglycerides, (D) alanine aminotransferase (ALT) and aspartate aminotransferase (AST), (E) alkaline phosphatase (alk phos) and total bilirubin in AAV-GFP and AAV-Cre injected mice (n=2). Student’s unpaired t test was not significant comparing any of the serum values between two groups. Reference ranges: BUN 18-29 mg/dL, albumin 2.5-4.8 g/dL, cholesterol 36-96 mg/dL, triglycerides 55-144 mg/dL, ALT 28-132 U/L, AST 59-347 U/L, alkaline phosphatase 52-209 U/L, total bilirubin 0.1-0.9 mg/dL. Note that albumin in Cre samples in panel B and total bilirubin in panel E have no error bars because the values were the same for both replicates. Hematoxylin and eosin (H&E) staining of liver section from AAV-GFP injected mice at 10X (F) and 20X (G) magnification and AAV-Cre injected mice at 10X (H) and 20X (I) magnification show intact tissue architecture, no fibrosis, and no inflammation. Arrows point to central veins and arrowheads indicate portal tracts. Scale bars represent 100 μm. (TIF) [file pone.0083723.s008.tif]
