# Supplementary material for: Function of GATA Factors in the Adult Mouse Liver
Source: PLoS One. 2013 Dec 18;8(12):e83723. doi: 10.1371/journal.pone.0083723 (PMC3867416; doi:10.1371/journal.pone.0083723)
Supplement: Table S1 — Genotyping primer sequences. Sequences for forward and reverse genotyping primers for each mouse strain. (PDF) [file pone.0083723.s009.pdf]

Genotyping primers **Table S1**

Primer name	Forward sequence	Reverse sequence
FOG1 ki/ki	CGCGCCGAGTGCCAAGCGCGCCGT	CCCTATCGCCGCACCATCTCGGAT
GATA4 fl/fl	CCCAGTAAAGAAGTCAGCACAAGGAA	AGACTATTGATCCCGGAGTGAACA
GATA6 fl/fl	GTGGTTGTAAGGCGGTTTGT	ACGCGAGCTCCAGAAAAAGT
