# Supplementary material for: Function of GATA Factors in the Adult Mouse Liver
Source: PLoS One. 2013 Dec 18;8(12):e83723. doi: 10.1371/journal.pone.0083723 (PMC3867416; doi:10.1371/journal.pone.0083723)
Supplement: Table S2 — RT-qPCR primer sequences. Sequences for forward and reverse RT-qPCR primers. (PDF) [file pone.0083723.s010.pdf]

RT-qPCR primers **Table S2**

Primer name	Forward sequence	Reverse sequence
Actin	ACACCCGCCACCAGTTC	TACAGCCCGGGGAGCAT
GAPDH	AGGTTGTCTCCTGCGACTTCA	CCAGGAAATGAGCTTGACAAA
GATA1	GCCCAAGAAGCGAATGATTG	GTGGTCGTTTGACAGTTAGTGCAT
GATA2	ACCACAAGATGAATGGACAGAA	GTCGTCTGACAATTTGCACAAC
GATA3	CTGGAGGAGGAACGCTAATG	TGCACCTTTTTGCACTTTTTC
GATA4	CGAGGGTGAGCCTGTATGTAA	CTGCTGTGCCCATAGTGAGAT
GATA5	CCGGAGCCTTCGACA	TCCACAGTTGACGCACT
GATA6	GACTGTCCTGTGCCAACTGTCA	TGGAGTTTCATATAGAGCCCGC
FOG1	CAGCAGCCAAACTTCCTCCA	GCGAGTGCTGTTGAAAGCCT
FOG2	GAACCTGCAAGCCCATTTGA	GCTTCTCGTTGCCTCCCAC
HNF4α	GGCATGAAGAAGGAAGCTGTCCAA	CTTTGCCCGAATGTCGCCATTGAT
Factor X	TGCTCCCTGGGAAAGGTGTGTTTA	AAATGAGTTTGCCCTCCGAGTCCT
Albumin	TTTCGCCGAGAAGCACACAAGAGT	AAGGCAATCAGGACTAGGCCTTTG
CD31	ACCTCCAACGAGAACTTTGTGC	CAATTTGAATCCGGACAGGATC
VE-cadherin	AAGATGCTGGCTGAGCTGTACG	GATCCAGGTTGCAATGAGGTTG
CD68	GCGCAGAATTCATCTCTTCGAG	AGCAGGTCAAGGTGAACAGCTG
CK7	AGGAGATCAACCGACGCAC	GTCTCGTGAAGGGTCTTGAGG
CK19	CTCGGATTGAGGAGCTGAAC	TCACGCTCTGGATCTGTGAC
Ppp4r4	TGGAGAAGGAGAAGCATCAGA	GAAATGGGCAAGTTCTTGGA
Stab2	TTCCCCTCACTCACGAACTT	TGATGGACAGGTCAGTCAGG
Arsa	ACGAGATCCATGGGGTCTTT	CATGACAGGCAGGATCTGAA
Amotl2	AAGGGCTCGTATCCAGTGAG	CGTCTCTGCTGCCATGTTT
Cyp7b1	TCTCTCTGCGGAAAGGAGAT	AAGCGATCGAACCTAAATTCC
Egfr	AGGCCATGAACATCACCTGT	GTTGTTCTCTCCCATGATGC
Inmt	AGAGCCAGGAGCCTACGACT	ACATCACACCTCAGGACACG
Prkd3	TGCGTTTATGTACCCACCAA	TGACTGAGAGATTTGTCAACACTG
Abhd2	TGAGGAAGAGAGCTGCATGA	GAATGGTTAGAAGGCTTTCGTG
Soat2	GGGTTCTTCTACCCGGTCAT	GGTCCACATCAGGATGTTCC
Cish	TGTGCATAGCCAAGACGTTC	CTCCGGCATCTTCTGTAGGT
Zfp36l1	CGCCACCATTTTTGACTTG	CCACTGCCTTCCTGTCCA
Gpr155	GCCCAGGAAGAAGAGCAGTA	CACAGGCAGCTGGAGAGATT
Ahctf1	TGAGCCTTTATTTCCTCCTCCT	GGGGAAACAACAAACTGAGAA
Ces1g	CCTGTTCCTGGACTTGATTG	AACAATTCCTGGGGTCTATCG
Cyp2u1	AGACGACTTCTGTCCTCATCG	CTGCATCAGGCTCACGAAC
Ces1e	CCAAGCTTCTCGTCAGAAATG	AAAGTTGGCCCAGAATTTCA
CD82	GTGCTGGTGTTGCTGTCATT	CAGTACTTGGGGACCTTGCT
Cyp2c29	GTTTGACCCTGGGCACTTT	GCCCTCTCCAGCACAAATC
Hsd17b2	CCATGTTCTCAACAATCATCAGA	TCTTTGGAAAAGTGGTCCAG
Abcg5	GGCTGCTTATTGGATCTGGA	GGCCGTAAAACTCATTGACC
Abcg8	GGGGCTGATGCAGATTCA	CAGGTCCATGGCACTGAT
Bhmt	CACCGGCTTCAGAAAAACAT	CGGAAGCTATTCGCAGATTC
Atg16l2	TGCAGTTCTGACTGGACCAA	TAGGCTGGTCTCCAGTTTCC
Slc44a1	TGTTCTTGTGTTTTGCCATTG	CGGCTTCTTTCATTGCTTTC
