# Supplementary material for: Function of GATA Factors in the Adult Mouse Liver
Source: PLoS One. 2013 Dec 18;8(12):e83723. doi: 10.1371/journal.pone.0083723 (PMC3867416; doi:10.1371/journal.pone.0083723)
Supplement: Table S3 — ChIP primer sequences. Sequences for forward and reverse ChIP-qPCR primers. (PDF) [file pone.0083723.s011.pdf]

ChIP-qPCR primers **Table S3**

Primer name	Forward sequence	Reverse sequence
Alb enh 1	CGAGATGGTACTTTGTGTCTCCTGC	GGCAGAGGACTGTATTGATCAG
Alb enh 2	CCCTTGAGCATGAGGTGACACTACT	AAAACTGGCCAAGGCAAACACGTC C
Alb neg 1	TCCTACTGCAGGGCTCTTGC	TGTAGCCTTGGGCTTGTGCT
Alb neg 2	GGCAAAATGAAGTGGGTAACCT	AGAGCCGGAGACGAAGAGG
FX prom 1	GGCCCTCTCAGCACTGGTAA	GAGTACGGTGATGACACTTGGCT
FX prom 2	GTGTGTTTGTCAGGTACCCACAG	TTACCAGTGCTGAGAGGGCC
FX neg 1	GCGTCATGGCCTTAGTTTCC	GTGAGATGGATGCCTGCCTAC
FX neg 2	TTTCCACAAGACCCAGGAGC	AGGCAGTTTGTGGCTGGATT
Peak 20, FOG site 2	GGACTCTGACCCATGGCTTC	CTCCCCAATCAGATAAAGGCC
Peak 14	CAGACACACAGAGAACACGCTG	GGAGGAAACCGGCTGGAG
Peak 34	AACTGGTGTCAGGTCGGGC	GGGCCTCGGATTTCCAACT
Peak 37	CCTTCTGCTATCAACCGGGA	CTGGCCCCGGCTTTATTT
Peak 38	TTGCTCAGCCTGTCTAGGGC	ACCAAGCAGGAAGCTCACAGA
Peak 5	GGGTCTCTGATAGATGGCTAGCTT	AACCCAGGCTTATCTCTAGCCTG
Peak 19, FOG site 1	TCTGTGCGCCTTTTGTTCTG	GCCTTATCAGCACGGCACAT
Peak 10	GCTTGGTTTGGAGAGTTGCC	AGGGTGTTTTGGAGGCTGC
Peak 33	CAACCTGCCGTCTTGATGTACA	GCTCCAAAGCTCTGGGCTG
Peak 27	CATCATGGCCTGTCCGTCT	GAGAGGCACATCAACCGTTGA
Peak 7	AGCTGCCACTCCCTGATTTG	GCGAGATAAAGCGGGCAAG
Peak 39	ACTGCCAGCTATTACCGAGACC	CCTTTAAATGCCTGCAAGCG
Peak 1	CATGGGAGTTGCTCTTCTTTGA	TCACCTCCATGCCTCAGTTTCT
Peak 31	CTGACGCCAGTGAGTCTGGA	CCTTGTTCCCCAATTTGCTG
Peak 3	GGAAGGAAGACGCTGGAGC	GCCCTTCCAGAACCCTGTTT
Peak 23	GGCTGCATCTTCTTTCCCAG	CGTCAGCTCTTCCACCCAGA
Peak 4	TTCCCCAGTCGATGATAGAAACA	GGGAGCCCACACCTCTTTGT
Peak 26	AAGCCACACTGCTCCGCTT	CGGGAGTTGCAGGTGATTTC
Peak 25	ACTTCGCCTGTGGATAGAGGG	CACGTGTATGTTCCCCAGCC
HNF4α prom	CGTATCCACCCACCTTGGG	CCGCCCGGTTATCTTATTGA
HNF4α end	GCCGGCTAACCTTGAGGATC	TGAAAGACCGCTGTCAGCAG
HNF6 body	TGCACACGTCCATTTGCTTT	AATGTCACCGGTCGGGATAA
Pppr4r ChIP	ACAACCTCTGCTCCCTGCAA	ATATGTGCCCTCGGCTATCAA
Arsa ChIP	GGATAGGGTGAGGCTGGAGAT	GCGCAGATAAGGATCGGGA
Amotl2 ChIP	AGGAAGTGGGCTGTTTTGCA	CACCGCCCTCCTCCCTAT
Cyp7b1 ChIP	CAGCGTCTGTCATGCTTTGG	GGCGCTGATAAGGATCTCTGG
Egfr ChIP	TGTTTCTCCAGCTAAGCACTATCTCA	ACACCCACAGTGTCACTGGGT
Inmt ChIP	AGAACCAGAACAGAAGTGCTCCC	TCTCTGGTCCTCCTACTTCCCC
Prkd3 ChIP	TGTGACCGCTTGCTTCCTTT	GCCACAGGCAAGATTAGAATGC

Stab2 ChIP	TAGGTCACCGCTGGTGGGT	TGATATATGTGGAAAGCCTTGACAC
Abhd2 ChIP	TATGTCAGTGGCCCAGGTTG	CCCATCTCTCTGCCATGATTC
Soat2 ChIP	GGCCTCAGTCCGAGATCACA	AGGTCAGTGATAGAAGCAAGATTG TAAT
Cish ChIP	ATGAGGCTTCCGGGAAGG	CGCCTCCTAATCTTTTGTCCG
Zfp36l1 ChIP	GCACGACAAGCTGATAGCACA	AGTTACAGGGCTATGAGTGACGG
Gpr155 ChIP	TGCAGGATCAGACTTTGGCC	GGTCAGGGACTCTGCACTCC
Ahctf1 ChIP	AGGGTCTCTCCGCCTCCTAG	ATGGGACTTGTAGTCTTTCGCC
Ces1g ChIP	GCCTGTGCCAGAGGCAGTTA	GCTGGATTTTACTGTGGGTCATT
Cyp2u1 ChIP	CATTATCAGTTTCCCCATCGATC	ATGACTTAACCAGTGGAGACAGGT
Ces1e ChIP	ATTGACCCTAACAAATATTGACCAGA	CCAGATAAGAGGGAGGACCTCA
CD82 ChIP	TCTCCTGTGTTGTAACCTTCTGTGA	GAATCCAGCAGTCCCCAAGTC
Cyp2c29 ChIP	TCATGGATAGAATTGTTCTGAATGCT	TCTCTTGCATTTTGTGCCCTT
Hsd17b2 ChIP	GCCAAGTGTTTCCTCATCCC	TGATAAGAAGGTGAGGAAGCCAG
Abcg5/8 ChIP	GGCAGAACACACGGTGTGTT	CCAGCATTCCTCTCTGGCAA
Bhmt ChIP	GCTGCTAGCTCGTCTGCACA	GGTCAGAGGCCTGCTATTGG
Atg16l2 ChIP	TGCCTCGGTAAACAGCAAAA	CCAACTCGAGGACCTCATCG
Slc44a1 ChIP	TCCTAACTTAGGCGCTGTGCTC	GACAGGAAAGCCTTCTGCCC
