# Supplementary material for: Function of GATA Factors in the Adult Mouse Liver
Source: PLoS One. 2013 Dec 18;8(12):e83723. doi: 10.1371/journal.pone.0083723 (PMC3867416; doi:10.1371/journal.pone.0083723)
Supplement: Table S4 — Genes up-regulated upon Gata4 excision that contain at least one GATA4 OS. “Start” and “end” denote genomic coordinates of GATA4 OS. (chr=chromosome, plus sign (+) represents upregulated). Note that some genes have more than one GATA4 OS. (PDF) [file pone.0083723.s012.pdf]

Upregulated genes from transcriptome of GATA4 excised hepatocytes Table S4

GATA4 ChIP-seq peaks within 10 kb upstream of TSS and 10 kb downstream of TTS

Chr	Start	End	Gene symbol	Accession #	Fold change	Gene name
chr15	100504392	100504892	Cela1/Ela1	NM_033612	+1.89	chymotrypsin-like elastase family, member 1
chr2	152906868	152907368	ccml2	NM_145536	+1.88	cerebral cavernous malformation 2-like
chrX	137067023	137067176	Tsc22d3	NM_010286	+1.88	TSC22 domain family, member 3
chrX	137102328	137102647	Tsc22d3	NM_010286	+1.88	TSC22 domain family, member 3
chr15	89306555	89307055	Arsa	NM_009713	+1.84	arylsulfatase A
chr6	55125442	55125942	Inmt	NM_009349	+1.74	indolethylamine N-methyltransferase
chr3	14861090	14861590	Car3	NM_007606	+1.73	carbonic anhydrase 3
chr10	86455278	86455778	Stab2	NM_138673	+1.72	stabilin 2
chr10	86453308	86453808	Stab2	NM_138673	+1.72	stabilin 2
chr11	98644529	98644764	Nr1d1	NM_145434	+1.69	Nuclear receptor subfamily 1, group D, member 1
chr14	31981624	31982124	Stab1	NM_138672	+1.64	stabilin 1
chrX	10079714	10080214	Tspan7	NM_019634	+1.60	tetraspanin 7
chr9	102624338	102624838	Amotl2	NM_019764	+1.59	angiomotin-like 2
chr9	102628538	102629038	Amotl2	NM_019764	+1.59	angiomotin-like 2
chr3	18109559	18110059	Cyp7b1	NM_007825	+1.56	cytocrhome p450, family 7, subfamily b, polypeptide 1
chr11	16700514	16701014	Egfr	NM_207655	+1.56	epidermal growth factor receptor
chr14	56204609	56204704	Fitm1	NM_026808	+1.56	Fat storage-inducing transmembrane protein 1
chr10	117166395	117166895	Slc35e3	NM_029875	+1.55	solute carrier family 35, member E3
chr12	104789622	104790122	Ppp4r4	NM_028980	+1.53	protein phosphatase 4, regulatory subunit 4
chr11	75281276	75281776	Tlcd2	NM_027249	+1.51	TLC domain containing 2
chr1	133860876	133861071	Slc45a3	NM_145977	+1.51	Solute carrier family 45, member 3
chr17	79383745	79384245	Prkd3	NM_029239	+1.50	protein kinase D3
