# Supplementary material for: Function of GATA Factors in the Adult Mouse Liver
Source: PLoS One. 2013 Dec 18;8(12):e83723. doi: 10.1371/journal.pone.0083723 (PMC3867416; doi:10.1371/journal.pone.0083723)
Supplement: Table S5 — Genes down-regulated upon Gata4 excision that contain at least one GATA4 OS. “Start” and “end” denote genomic coordinates of GATA4 OS. (chr=chromosome, negative sign (-) represents downregulated). Note that some genes have more than one GATA4 OS. (PDF) [file pone.0083723.s013.pdf]

Downregulated genes from transcriptome of GATA4 excised hepatocytes Table S5

GATA4 ChIP-seq peaks within 10 kb upstream of TSS and 10 kb downstream of TTS

**Chr**	**Start**	**End**	**Gene symbol**	**Accession #**	**Fold change**	**Gene name**
chr1	181733822	181734322	Ahctf1	NM_026375	-1.53	AT hook containing transcription factor 1
chr11	101329652	101332620	Rnd2	NM_009708	-1.54	Rho family GTPase 2
chr19	31961726	31962226	A1cf	NM_001081074	-1.55	APOBEC1 complementation factor
chr19	40314052	40314552	Pdlim1	NM_016861	-1.62	PDZ and LIM domain 1 (elfin)
chr1	193578002	193578502	Lpgat1	NM_001134829	-1.64	lysophosphatidylglycerol acyltransferase 1
chr15	80079191	80079300	Atf4	NM_009716	-1.74	Activating transcription factor 4
chr2	73223630	73224130	Gpr155	NM_001190297.2	-1.78	G protein-coupled receptor 155
chr15	101979940	101980440	Soat2	NM_146064	-1.79	sterol O-acyltransferase 2
chr15	101980658	101981158	Soat2	NM_146064	-1.79	sterol O-acyltransferase 2
chr7	86456263	86456763	Abhd2	NM_018811	-1.84	abhydrolase domain containing 2
chr7	86503340	86503840	Abhd2	NM_018811	-1.84	abhydrolase domain containing 2
chr7	86508148	86508648	Abhd2	NM_018811	-1.84	abhydrolase domain containing 2
chr12	81203999	81204499	Zfp36l1	NM_007564	-1.90	zinc finger protein 36, C3H type-like 1
chr8	95856550	95857050	Ces1g	NM_021456	-2.15	carboxylesterase 1G
chr11	115335091	115335591	Slc16a5	NM_001080934	-2.66	solute carrier family 16, member 5
chr9	107198878	107199378	Cish	NM_009895	-4.34	cytokine inducible SH2-containing protein
