# Supplementary material for: Function of GATA Factors in the Adult Mouse Liver
Source: PLoS One. 2013 Dec 18;8(12):e83723. doi: 10.1371/journal.pone.0083723 (PMC3867416; doi:10.1371/journal.pone.0083723)
Supplement: Table S6 — Differentially expressed genes upon Gata4,6 double excision that contain at least one GATA4 OS. “Start” and “end” denote genomic coordinates of GATA4 OS. (chr=chromosome, positive sign (+) or negative sign (-) represent upregulated or downregulated, respectively). Note that some genes have more than one GATA4 OS. (PDF) [file pone.0083723.s014.pdf]

**Differentially expressed genes from transcriptome of GATA4,6 excised hepatocytes** Table S6

**GATA4 ChIP-seq peaks within 10 kb upstream of TSS and 10 kb downstream of TTS**

| Chr | Start | End | Gene symbol | Accession # | Fold change | Gene name |
|---|---|---|---|---|---|---|
| chrX | 10079896 | 10080031 | Tspan7 | NM_019634 | +2.03 | tetraspanin 7 |
| chr8 | 95757995 | 95758180 | Ces1e | NM_133660 | -1.51 | carboxyesterase 1E |
| chr2 | 93285527 | 93285699 | CD82 | NM_007656 | -1.55 | CD82 antigen |
| chr2 | 93294989 | 93295273 | CD82 | NM_007656 | -1.55 | CD82 antigen |
| chr7 | 30023192 | 30023512 | 2200002D01Rik | NM_028179 | -1.58 | 2200002D01Rik RIKEN cDNA 2200002D01 gene |
| chr7 | 30038981 | 30039179 | 2200002D01Rik | NM_028179 | -1.58 | 2200002D01Rik RIKEN cDNA 2200002D01 gene |
| chr13 | 34058505 | 34058760 | Nqo2 | NM_020282 | -1.59 | NAD(P)H dehydrogenase, quinone 2 |
| chr13 | 34059584 | 34059727 | Nqo2 | NM_020282 | -1.59 | NAD(P)H dehydrogenase, quinone 2 |
| chr11 | 79972651 | 79972815 | Adap2 | NM_172133 | -1.60 | ArfGAP with dual PH domains 2 |
| chr1 | 173355242 | 173355438 | Tstd1 | NM_001164525 | -1.60 | thiosulfate sulfurtransferase (rhodanese)-like domain containing 1 |
| chr17 | 85082225 | 85082510 | Abcg5 | NM_031884 | -1.65 | ATP-binding cassette, sub-family G (WHITE), member 5 |
| chr4 | 53605353 | 53605478 | Slc44a1 | NM_133891 | -1.66 | solute carrier family 44, member 1 |
| chr4 | 53605353 | 53605478 | Slc44a1 | NM_133891 | -1.66 | solute carrier family 44, member 1 |
| chr7 | 4581868 | 4582089 | Tmem86b | NM_023440 | -1.66 | transmembrane protein 86B |
| chr5 | 138362926 | 138363246 | Cyp3a13 | NM_007819 | -1.70 | cytochrome P450, family 3, subfamily a, polypeptide 13 |
| chr7 | 108459316 | 108459522 | Atg16l2 | NM_001111111 | -1.72 | Atg16l2 autophagy related 16-like 2 |
| chr9 | 122754860 | 122755053 | Zfp445 | NM_173364 | -1.72 | zinc finger protein 445 |
| chr17 | 85082225 | 85082510 | Abcg8 | NM_026180 | -1.73 | ATP-binding cassette, sub-family G (WHITE), member 8 |
| chr3 | 131015374 | 131015495 | Cyp2u1 | NM_027816 | -1.73 | cytochrome P450, family 2, subfamily u, polypeptide 1 |
| chr8 | 120219653 | 120219852 | Hsd17b2 | NM_008290 | -1.83 | hydroxysteroid (17-beta) dehydrogenase 2 |
| chr8 | 120224683 | 120225003 | Hsd17b2 | NM_008290 | -1.83 | hydroxysteroid (17-beta) dehydrogenase 2 |
| chr8 | 120238674 | 120238823 | Hsd17b2 | NM_008290 | -1.83 | hydroxysteroid (17-beta) dehydrogenase 2 |
| chr19 | 3709053 | 3709211 | 1810055G02Rik | NM_028077 | -1.85 | 1810055G02Rik RIKEN cDNA 1810055G02 gene |
| chr13 | 94407548 | 94407868 | Bhmt | NM_016668 | -1.88 | betaine-homocysteine methyltransferase |
| chr8 | 95856640 | 95856960 | Ces1g | NM_021456 | -1.89 | carboxylesterase 1G |
| chr19 | 39360278 | 39360369 | Cyp2c29 | NM_007815 | -2.23 | cytochrome P450, family 2, subfamily c, polypeptide 29 |
| chr1 | 72219981 | 72220253 | Mreg | NM_001005423 | -2.34 | melanoregulin |
| chr1 | 72226679 | 72226779 | Mreg | NM_001005423 | -2.34 | melanoregulin |
| chr2 | 73223720 | 73224040 | Gpr155 | NM_001276443 | -2.38 | G protein-coupled receptor 155 |
| chr15 | 101980077 | 101980302 | Soat2 | NM_146064 | -2.39 | sterol O-acyltransferase 2 |
| chr15 | 101980853 | 101980963 | Soat2 | NM_146064 | -2.39 | sterol O-acyltransferase 2 |
